# Supplementary material for: International assessment of the link between COVID-19 related attitudes, concerns and behaviours in relation to public health policies: optimising policy strategies to improve health, economic and quality of life outcomes (the iCARE Study)
Source: BMJ Open. 2021 Mar 11;11(3):e046127. doi: 10.1136/bmjopen-2020-046127 (PMC7956731; doi:10.1136/bmjopen-2020-046127)
Supplement: Supplementary data [file bmjopen-2020-046127supp001.pdf]

**Supplementary Material**

**iCARE Study team**

**Lead investigators**: Kim L. Lavoie, PhD, University of Quebec at Montreal (UQAM) and CIUSSS-NIM, CANADA; Simon L. Bacon, PhD, Concordia University and CIUSSS-NIM, CANADA.

**Collaborators (in alphabetical order: Country then investigator)**: ABU DHABI: Zahir Vally, PhD, United Arab Emirates University; AUSTRALIA: Jacqueline Boyle, PhD, Monash University; Joanne Enticott, PhD, Monash University; Shajedur Rahman Shawon, PhD, Centre for Big Data Research in Health, UNSW Medicine; Helena Teede, MD, Monash University; AUSTRIA: Alexandra Kautzky-Willer, MD, Medizinische Universität Wien; BANGLADESH: Arobindu Dash, MS, International University of Business, Agriculture & Technology; BRAZIL: Marilia Estevam Cornelio, PhD, University of Campinas; Marlus  Karsten, Universidade do Estado de Santa Catarina - UDESC; Darlan Lauricio Matte, PhD, Universidade do Estado de Santa Catarina - UDESC; CANADA: Ahmed Abou-Setta, PhD, University of Manitoba; Shawn Aaron, PhD, Ottawa Hospital Research Institute; Angela Alberga, PhD, Concordia University; Tracie Barnett, PhD, McGill University; Silvana Barone, MD, Université de Montréal; Ariane Bélanger-Gravel, PhD, Université Laval; Sarah Bernard, PhD, Université Laval; Lisa Maureen Birch, PhD, Université Laval; Susan Bondy, PhD, University of Toronto - Dalla Lana School of Public Health; Linda Booij, PhD, Concordia University; Roxane Borgès Da Silva, PhD, Université de Montréal; Jean Bourbeau, MD, McGill University; Rachel Burns, PhD, Carleton University; Tavis Campbell, PhD, University of Calgary; Linda Carlson, PhD, University of Calgary; Kim Corace, PhD, University of Ottawa; Olivier Drouin, MD, CHU Sainte-Justine/Université de Montréal; Francine Ducharme, MD, Université de Montréal; Mohsen Farhadloo, Concordia University; Carl Falk, PhD, McGill University; Richard Fleet MD, PhD, Université Laval; Michel Fournier, MSc, Direction de la Santé Publique de Montréal; Gary Garber, MD, University of Ottawa/Public Health Ontario; Lise Gauvin, PhD, Université de Montréal; Jennifer Gordon, PhD, University of Regina; Roland Grad, MD, McGill University; Samir Gupta, MD, University of Toronto; Kim Hellemans, PhD, Carleton University; Catherine Herba PhD, UQAM; Heungsun Hwang, PhD, McGill University; Keven Joyal-Desmarais, PhD, Concordia University; Lisa Kakinami, PhD, Concordia University; Eric Kennedy, PhD, York University; Sunmee Kim, PhD, University of Manitoba; Joanne Liu, PhD, McGill University; Sandra Pelaez, PhD, Université de

Montréal; Louise Pilote, MD, McGill University; Paul Poirier, MD, Université Laval; Justin Presseau, PhD, University of Ottawa; Eli Puterman, PhD, University of British Columbia; Joshua Rash, PhD, Memorial University; Paula AB Ribeiro, PhD, MBMC; Mohsen Sadatsafavi, PhD, University of British Columbia; Paramita Saha Chaudhuri, PhD, McGill University; Jovana Stojanovic, PhD, Concordia University; Eva Suarthana, MD, PhD, Université de Montréal / McGill University; Michael Vallis, PhD, Dalhousie University; CHILE: Nicolás Bronfman Caceres, PhD, Universidad Andrés Bello; Manuel Ortiz, PhD, Universidad de La Frontera; Paula Beatriz Repetto, PhD, Universidad Católica de Chile; COLOMBIA: Mariantonia Lemos-Hoyos, PhD, Universidad EAFIT; CYPRUS: Angelos Kassianos, PhD, University of Cyprus; DENMARK: Naja Hulvej Rod, PhD, University of Copenhagen; FRANCE: Mathieu Beraneck, PhD, Université de Paris; CNRS; Gregory Ninot, PhD, Université de Montpellier; GERMANY: Beate Ditzen, PhD, Heidelberg University; Thomas Kubiak, PhD, Mainz University; GHANA: Sam Codjoe MPhil,MSc, University of Ghana; Lily Kpobi, PhD, University of Ghana; Amos Laar, PhD, University of Ghana; INDIA: Naorem Kiranmala Devi, PhD, University of Delhi; Sanjenbam Meitei, PhD, Manipur University; Suzanne Tanya Nethan, MDS,  ICMR-National Institute of Cancer Prevention & Research; Lancelot Pinto, MD, PhD, Hinduja Hospital and Medical Research Centre; Kallur Nava Saraswathy, PhD, University of Delhi; Dheeraj Tumu, MD, World Health Organization (WHO); INDONESIA: Silviana Lestari, MD, PhD, Universitas Indonesia; Grace Wangge, MD, PhD, SEAMEO Regional Center for Food and Nutrition; IRELAND: Molly Byrne, PhD, National University of Ireland, Galway; Hannah Durand, PhD, National University of Ireland, Galway; Jennifer McSharry, PhD, National University of Ireland, Galway; Oonagh Meade, PhD, National University of Ireland, Galway; Gerry Molloy, PhD, National University of Ireland, Galway; Chris Noone, PhD, National University of Ireland, Galway; ISRAEL: Hagai Levine, MD, Hebrew University; Anat Zaidman-Zait, PhD, Tel-Aviv University; ITALY: Stefania Boccia, PhD, Università Cattolica del Sacro Cuore; Ilda Hoxhaj, MD, Università Cattolica del Sacro Cuore, Stefania Paduano, MSc, PhD, University of Modena and Reggio Emilia; Valeria Raparelli, PhD, Sapienza - University of Rome; Drieda Zaçe, MD, MSc, PhDc, Università Cattolica del Sacro Cuore; JORDAN: Ala'S Aburub, PhD, Isra University; KENYA: Daniel Akunga, PhD, Kenyatta University; Richard Ayah, PhD, University of Nairobi, School Public Health; Chris Barasa, MPH, University of Nairobi, School Public Health; Pamela Miloya Godia, PhD, University of Nairobi; Elizabeth W. Kimani-Murage, PhD, African Population and Health Research Center; Nicholas Mutuku, PhD, University of Kenya; Teresa Mwoma, PhD, Kenyatta University; Violet Naanyu, PhD, Moi University; Jackim Nyamari, PhD, Kenyatta University; Hildah Oburu, PhD, Kenyatta University; Joyce Olenja, PhD, University of Nairobi;

Dismas Ongore, PhD, University of Nairobi; Abdhalah Ziraba, PhD, African Population and Health Research Center; MALAWI: Chiwoza Bandawe, PhD, University of Malawi; MALAYSIA: Loh Siew Yim, PhD, Faculty of medicine, University of Malaya; NEW ZEALAND: Andrea Herbert, PhD, University of Canterbury; Daniela Liggett, PhD, University of Canterbury; NIGERIA: Ademola Ajuwon, PhD, University of Ibadan; PAKISTAN: Nisar Ahmed Shar, PhD, CoPI-National Center in Big Data & Cloud Computing; Bilal Ahmed Usmani, PhD, NED University of Engineering and Technology; PERU: Rosario Mercedes Bartolini Martínez, PhD, Instituto de Investigacion Nutricional; Hilary Creed-Kanashiro, M.Phil., Instituto de Investigacion Nutricional; PORTUGAL: Paula Simão, MD, S. Pneumologia de Matosinhos; RWANDA: Pierre Claver Rutayisire, PhD, University Rwanda; SAUDI ARABIA: Abu Zeeshan Bari, PhD, Taibah University; SLOVAKIA: Iveta Nagyova, PhD, PJ Safarik University - UPJS; SOUTH AFRICA: Jason Bantjes, PhD, University of Stellenbosch; Brendon Barnes, PhD, University of Johannesburg; Bronwyne Coetzee, PhD, University of Stellenbosch; Ashraf Khagee, PhD, University of Stellenbosch; Tebogo Mothiba, PhD, University of Limpopo; Rizwana Roomaney, PhD, University of Stellenbosch; Leslie Swartz, PhD University of Stellenbosch; SOUTH KOREA: Juhee Cho, PhD, Sungkyunkwan University; Man-gyeong Lee, PhDc, Sungkyunkwan University; SWEDEN: Anne Berman, PhD, Karolinska Institutet; Nouha Saleh Stattin, MD, Karolinska Institutet; SWITZERLAND: Susanne Fischer, PhD, University of Zurich; TAIWAN: Debbie Hu, MD, MSc, Tainan Municipal Hospital; TURKEY: Yasin Kara, MD, Kanuni Sultan Süleyman Training and Research Hospital, Istanbul; Ceprail Şimşek, MD Health Science University; Bilge Üzmezoğlu, MD, University of Health Science; UGANDA: John Bosco Isunju, PhD, Makerere University School of Public Health; James Mugisha, PhD, University of Uganda; UK: Lucie Byrne-Davis, PhD, University of Manchester; Paula Griffiths, PhD, Loughborough University; Joanne Hart, PhD, University of Manchester; Will Johnson, PhD, Loughborough University; Susan Michie, PhD, University College London; Nicola Paine, PhD, Loughborough University; Emily Petherick, PhD, Loughborough University; Lauren Sherar, PhD, Loughborough University; USA: Robert M. Bilder, PhD, ABPP-CN, University of California, Los Angeles; Matthew Burg, PhD, Yale; Susan Czajkowski, PhD, NIH - National Cancer Institute; Ken Freedland, PhD, Washington University; Sherri Sheinfeld Gorin, PhD, University of Michigan; Alison Holman, PhD, University of California, Irvine; Jiyoung Lee, PhD, University of Alabama; Gilberto Lopez ScD, MA, MPH, Arizona State University and University of Rochester Medical Center; Sylvie Naar, PhD, Florida State University; Michele Okun, PhD, University of Colorado, Colorado Springs; Lynda Powell, PhD, Rush University; Sarah Pressman, PhD, University of California, Irvine; Tracey Revenson, PhD, University of New York City; John Ruiz, PhD, University of

Arizona; Sudha Sivaram, PhD, NIH, Center for Global Health; Johannes Thrul, PhD, Johns Hopkins; Claudia Trudel-Fitzgerald, PhD, Harvard T.H. Chan School of Public Health; Abehaw Yohannes, PhD, Azusa Pacific University.

**Students (in alphabetical order):** AUSTRALIA: Rhea Navani, BSc, Monash University; Kushnan Ranakombu, PhD, Monash University; BRAZIL: Daisuke Hayashi Neto, Unicamp; CANADA: Tair Ben-Porat, PhD, Tel Aviv University; Anda Dragomir, University of Quebec at Montreal (UQAM) and CIUSSS-NIM; Amandine Gagnon-Hébert, BA, UQAM; Claudia Gemme, MSc, UQAM; Vincent Gosselin Boucher, University of Quebec at Montreal (UQAM) and CIUSSS-NIM; Mahrukh Jamil, Concordia University and CIUSSS-NIM; Lisa Maria Käfer, McGill University; Ariany Marques Vieira, MSc, Concordia University; Tasfia Tasbih, Concordia University and CIUSSS-NIM; Maegan Trottier, University of Lethbridge; Robbie Woods, MSc, Concordia University; Reyhaneh Yousefi, Concordia University and CIUSSS-NIM; FRANCE: Tamila Roslyakova, Université de Montpellier; GERMANY: Lilli Priesterroth, Mainz University; ISRAEL: Shirly Edelstein, Hebrew University-Hadassah School of Public Health; Tanya Goldfrad, Hebrew University-Hadassah School of Public Health; Ruth Snir, Hebrew University-Hadassah School of Public Health; Yifat Uri, Hebrew University-Hadassah School of Public Health; NEW ZEALAND: Mohsen Alyami, University of Auckland; NIGERIA: Comfort Sanuade; SERBIA: Katarina Vojvodic, University of Belgrade.

**Community Participants:** CANADA: Olivia Crescenzi; Kyle Warkentin; DENMARK: Katya Grinko; INDIA: Lalita Angne; Jigisha Jain; Nikita Mathur, Syncorp Clinical Research; Anagha Mithe; Sarah Nethan, Community Empowerment Lab.
