# Supplementary material for: International assessment of the link between COVID-19 related attitudes, concerns and behaviours in relation to public health policies: optimising policy strategies to improve health, economic and quality of life outcomes (the iCARE Study)
Source: BMJ Open. 2021 Mar 11;11(3):e046127. doi: 10.1136/bmjopen-2020-046127 (PMC7956731; doi:10.1136/bmjopen-2020-046127)
Supplement: Author's manuscript [file bmjopen-2020-046127.draft_revisions.pdf]

BMJ Open

BMJ Open is committed to open peer review. As part of this commitment we make the peer review history of every article we publish publicly available.

When an article is published we post the peer reviewers' comments and the authors' responses online. We also post the versions of the paper that were used during peer review. These are the versions that the peer review comments apply to.

The versions of the paper that follow are the versions that were submitted during the peer review process. They are not the versions of record or the final published versions. They should not be cited or distributed as the published version of this manuscript.

BMJ Open is an open access journal and the full, final, typeset and author-corrected version of record of the manuscript is available on our site with no access controls, subscription charges or pay-per-view fees (http://bmjopen.bmj.com).

If you have any questions on BMJ Open's open peer review process please email info.bmjopen@bmj.com

BMJ Open

An international assessment of the link between COVID-19-related attitudes, concerns and behaviours in relation to public health policies: Optimising policy strategies to improve health, economic and quality of life outcomes (the iCARE Study). Protocol Paper

Journal:	*BMJ Open*
Manuscript ID	bmjopen-2020-046127
Article Type:	Protocol
Date Submitted by the Author:	21-Oct-2020
Complete List of Authors:	Bacon, Simon; Concordia University, Exercise Science<br>Lavoie, Kim; Université du Québec à Montréal, Psychology; Hôpital du Sacré-Coeur de Montréal, Axe de recherche en pneumologie
Keywords:	COVID-19, Health policy < HEALTH SERVICES ADMINISTRATION & MANAGEMENT, PREVENTIVE MEDICINE, PUBLIC HEALTH

SCHOLARONE™
Manuscripts

BMJ

I, the Submitting Author has the right to grant and does grant on behalf of all authors of the Work (as defined in the below author licence), an exclusive licence and/or a non-exclusive licence for contributions from authors who are: i) UK Crown employees; ii) where BMJ has agreed a CC-BY licence shall apply, and/or iii) in accordance with the terms applicable for US Federal Government officers or employees acting as part of their official duties; on a worldwide, perpetual, irrevocable, royalty-free basis to BMJ Publishing Group Ltd ("BMJ") its licensees and where the relevant Journal is co-owned by BMJ to the co-owners of the Journal, to publish the Work in this journal and any other BMJ products and to exploit all rights, as set out in our licence.

The Submitting Author accepts and understands that any supply made under these terms is made by BMJ to the Submitting Author unless you are acting as an employee on behalf of your employer or a postgraduate student of an affiliated institution which is paying any applicable article publishing charge ("APC") for Open Access articles. Where the Submitting Author wishes to make the Work available on an Open Access basis (and intends to pay the relevant APC), the terms of reuse of such Open Access shall be governed by a Creative Commons licence – details of these licences and which Creative Commons licence will apply to this Work are set out in our licence referred to above.

Other than as permitted in any relevant BMJ Author's Self Archiving Policies, I confirm this Work has not been accepted for publication elsewhere, is not being considered for publication elsewhere and does not duplicate material already published. I confirm all authors consent to publication of this Work and authorise the granting of this licence.

For peer review only

Title: An international assessment of the link between COVID-19-related attitudes, concerns and behaviours in relation to public health policies: Optimising policy strategies to improve health, economic and quality of life outcomes (the iCARE Study). Protocol Paper.

Brief title: The iCARE study: Protocol paper

Authors: Simon L. Bacon, PhD,[a,b] Kim L. Lavoie, PhD,[a,c] for the iCARE study team*

Word count: 2,335

Author affiliations:

a. Montreal Behavioural Medicine Centre (MBMC), Centre de recherche CIUSSS-NIM, 5400 Gouin West Blvd., Montreal, Quebec, H4J 1C5, Canada

b. Department of Health, Kinesiology, and Applied Physiology, Concordia University, Montreal, Canada

c. Department of Psychology, Université du Québec à Montréal (UQAM), Montreal, Canada

* The complete list of iCARE Study collaborators appears in the Supplementary Material.

Contributors statement: Both authors (SLB and KLL) contributed equally to the manuscript including: contributing substantially to conception and design; drafting the article and revising it critically for important intellectual content; providing final approval of the version to be published; and acting as guarantors of the work.

Funding statement: The primary source of funding for the iCARE study has been primarily through re-directed funding associated with Montreal Behavioural Medicine Centre, including funds from a Canadian Institutes of Health Research-Strategy for Patient Oriented Research Mentoring Chair (SMC-151518, PI: Dr. Simon L. Bacon), a Fonds de Recherche du Québec: Santé Chair (251618, PI: Dr. Simon L. Bacon), a UQAM Research Chair (1471, PI: Dr. Kim L Lavoie), and Fonds de Recherche du Québec: Santé Senior Research Award (34757, PI: Dr. Kim L Lavoie). The Canadian representative sampling will be funded by the Canadian Institutes of Health Research (MS3-173099, PI: Simon L. Bacon) and the Fonds de Recherche du Québec: Société et Culture (2019-SE1-252541, PI: Dr. Simon L. Bacon). The

For peer review only - http://bmjopen.bmj.com/site/about/guidelines.xhtml
For peer review only

Australian representative sampling was funded by Monash University and indirectly by the National Health and Medical Research Council and the Medical Research Future Fund (2579, PI: Dr. Helena Teede). The Irish representative sampling was funded by the Health Research Board and the Irish Research Council (COV19-2020-097, PI: Dr. Gerard J. Molloy). The UK representative sampling was funded by CALIBRE research funding, provided by Loughborough University, UK (5705, PI: Dr. Nicola J. Paine). None of the funders were involved in the study design.

Study registration: N/A

Competing interests:

Dr. Bacon has received consultancy fees from Merck for the development of behavior change continuing education modules, speaker fees from Novartis and Janssen, and has served on advisory boards for Bayer, Sanofi, and Sojecci Inc, none of which are related to the current article.

Dr. Lavoie has served on the advisory board for Schering-Plough, Takeda, AbbVie, Almirall, Janssen, GSK, Boehringer Ingelheim (BI), and Sojecci Inc, and has received sponsorship for investigator-generated research grants from GlaxoSmithKline (GSK) and AbbVie, speaker fees from GSK, Astra-Zeneca, Astellas, Novartis, Takeda, AbbVie, Merck, Boehringer Ingelheim, Bayer, Pfizer and Air Liquide, and support for educational materials from Merck, none of which are related to the current article.

Address for correspondence:

Simon L. Bacon, PhD, FTOS, FCCS, FABMR

Montreal Behavioral Medicine Centre

CIUSSS-NIM

Email: simon.bacon@concordia.ca

Twitter: @sbacon20, @mbmc_cmcm

For peer review only

For peer review only - http://bmjopen.bmj.com/site/about/guidelines.xhtml

Abstract:

Introduction: In the context of a highly contagious virus with no vaccine and no cure, the key to slowing the spread of the COVID-19 disease and successfully transitioning through the phases of the pandemic, is public adherence to rapidly evolving behaviour-based public health policies. The overall objective of the iCARE Study is to assess public awareness, attitudes, concerns, and behavioural responses to COVID-19 public health policies, and their impacts, on people around the world, and to link behavioural survey data with policy, mobility, and case data to provide behavioural science, data-driven recommendations to governments on how to optimise current policy strategies to reduce the impact of the COVID-19 pandemic.

Methods and analyses: The iCARE study (www.mbmc-cmcm.ca/covid19) utilises a multiple cross-sectional survey design to capture self-reported information on a variety of COVID-19 related variables from individuals around the globe. Survey data is captured using two data capture methods, convenience and representative sampling. This data is then coupled to open access data for policies, cases, and population movement.

Ethics and Dissemination: The primary ethical approval was obtained from the co-ordinating site, the CIUSSS-NIM (REB#: 2020-2099 / 03-25-2020). This study will provide high-quality, accelerated and real-time evidence to help us understand the effectiveness of evolving country-level policies and communication strategies to reduce the spread of the COVID-19. Due to the urgency of the pandemic, results will be disseminated in a variety of ways, including policy briefs, social media posts, press releases, and through regular scientific methods.

Registration: N/A

Keywords: Evidence-based policies; Behaviour change; COVID-19

For peer review only - http://bmjopen.bmj.com/site/about/guidelines.xhtml

Strengths and limitations of this study

- This is a large, international study that has data captured from over 150 countries.
- The survey data that is being captured was constructed around well recognised behavioural theories and frameworks.
- The study is primarily being conducted online which limits some of the generalisability of the data that is available, especially in lower and middle income countries.
- The primary data capture method is through snowball sampling which is likely to create some bias in the sample. However, some of this can be adjusted using weightings from the representative samples that are being collected.
- A key strength of the study is that it has been developed to provide constructive policy and communication data which can be quickly implemented by governments to improve adherence to COVID-19 mitigation methods.

For peer review only

For peer review only - http://bmjopen.bmj.com/site/about/guidelines.xhtml

Abbreviations:

CIUSSS-NIM – Centre intégré universitaire de santé et de services sociaux du Nord-de-l'Île-de-Montréal

COM-B – Capability, Opportunity, Motivation-Behaviour Model

iCARE – International assessment of the link between COVID-19-related attitudes, concerns and behaviours in relation to public health policies

IGLS – Iterative generalized least squares

LMIC – Low- and Middle-income countries

MBMC – Montreal Behavioural Medicine Centre

MCMC – Markov chain Monte Carlo

OxCGRT – Oxford COVID-19 Government Response Tracker

UQAM – Université du Québec à Montréal

For peer review only

For peer review only - http://bmjopen.bmj.com/site/about/guidelines.xhtml

Introduction:

With no current vaccine nor cure, the key to slowing the spread of COVID-19 and successfully transitioning through the phases of the pandemic, is ***public adherence*** to unprecedented and rapidly evolving **behaviour-based** public health policies (1, 2). To date, adherence to these policies has been critical to reducing the spread of COVID-19 and have ranged from personal hygiene measures (e.g., hand washing) to strict lockdown measures (e.g., business and school closures) (3-5). However, adherence to most of these policies requires making behavioural changes that may come with significant personal, social and economic costs, which may undermine their impact (6). For example, despite public health messages promoting the 'advantages' of adhering to COVID-19 mitigation measures, adherence to policies that may come with high personal costs (i.e., physical distancing) have been much poorer (54%) than for other 'less costly' behaviours like hand washing (90%) (7). Further, as we look towards relaxing lockdown measures, people's willingness to adhere to changing government recommendations (e.g., school and store reopening's) will also be critical for re-engaging the economy whilst minimising the potential for future waves of the pandemic. Unfortunately, policy variations between and within countries, have created public confusion and uncertainty about government policy motives (8). In addition, governments have predominantly designed policies based on how they believe people 'should' behave and have ascribed little consideration to what we know about how people actually behave (9, 10).

Decades of behavioural science research has revealed that human behaviour is predictable and modifiable (11). Multiple factors are likely to predict why people adhere (or not) to various public health measures, which, in the context of COVID-19, can be defined using two related behaviour prediction models: 1) *The Capability, Opportunity, Motivation-Behaviour (COM-B) Model* (2, 12), which predicts that behaviour change depends on: awareness of prevention measures and the ability to enact them (capability), the belief that measures are personally relevant and important (motivation), and having the social and environmental resources required to adopt the behaviour (opportunity) (see **Figure 1a**); and 2) *The Health Beliefs Model* (13, 14), which posits that in adopting disease prevention measures, a person's belief in the personal threat(s) posed by the disease, together with a person's belief in the importance and effectiveness of recommended behaviours, will predict the likelihood a person adopting (or not) a particular behaviour (**Figure 1b**). In the context of this unprecedented health, social, and economic crisis, where the global need for adherence to

For peer review only - http://bmjopen.bmj.com/site/about/guidelines.xhtml

rapidly evolving public health policies has never been greater, our understanding of the determinants of adherence at each phase of the pandemic, and as a function of various policies, is critical for effective policy planning, communication, and effectiveness.

Insert Figure 1 about here

The overall goal of the iCARE Study is to assess public awareness, attitudes, concerns, and behavioural responses to COVID-19 public health policies, and their impacts, on people around the world (www.mbmc-cmcm.ca/covid19), and to link behavioural survey data with policy, mobility, and case data to provide behavioural science, data-driven recommendations to governments on how to optimise current policy strategies to reduce the impact of the COVID-19 pandemic worldwide. Specifically, we will address the following:

1) What are the **key individual characteristics** (e.g., sociodemographic; psychological; behavioural; physical/mental health; and economic) that **are associated with adherence** to major COVID-19 public health policies *in general* and by *country*?
2) To what extent are COVID-19 **attitudes, beliefs and concerns associated with adherence**, and how does this vary across key subgroups?
3) What are the short- and medium-term **impacts of COVID-19 and its public health policies**, and how do they vary as a function of key individual characteristics in *general* and by *country*?
4) Which policies and strategies are associated with better (and worse) adherence, are most (and least) effective at reducing infection rates, and positively impact economic growth (where appropriate)? As well as, identifying in whom these polices and strategies worked (and did not work).
5) The development of **behavioural science, data-driven, tailored recommendations,** that governments could use to optimise policy and communication strategies to improve adherence, as well as, health, economic, and quality of life outcomes.

For peer review only - http://bmjopen.bmj.com/site/about/guidelines.xhtml

Methods and analysis

Study design:

The iCARE Study is a Canadian-led, ongoing, multi-wave international study involving the collaboration of more than **150 international researchers** from over **40 countries** (see Supplementary Material). It utilises a multiple cross-sectional survey design (each approximately 5 weeks apart) to capture self-reported information on a variety of COVID-19 related variables from individuals around the globe. Survey data is captured using two data capture methods, convenience and representative sampling (see details below). This data is then coupled to open access data for policies, cases, and population movement. The study is managed by the Montreal Behavioural Medicine Centre (MBMC: a joint Centre intégré universitaire de santé et de services sociaux du Nord-de-l'Île-de-Montréal (CIUSSS-NIM) / Université du Québec à Montréal (UQAM) / Concordia University academic research and training centre).

Patient and Public Involvement (PPI) Statement

Given the significance and broad impact of the COVID-19 pandemic PPI is crucial for effective research in this area. More importantly, given the global nature of the iCARE study it has been critical to have individuals from multiple settings included in the development of the various elements and items in the survey. To this end, we consulted with over 150 collaborators from more than 40 countries including researchers, clinicians, students, and members of the general public in the development and design of the iCARE study (see Supplementary Material for the iCARE team). In addition, throughout our data analysis process we have engaged critical end users, including government officials, the public, the news media, in defining areas that need critical input for which the iCARE study is able to address.

The iCARE survey:

The core elements of the survey assess the following domains:

- awareness of local COVID-19 public health policies
- attitudes/beliefs about local COVID-19 policies
- behavioural responses to local COVID-19 policies
- perceived concerns about COVID-19

For peer review only - http://bmjopen.bmj.com/site/about/guidelines.xhtml

- the impacts of COVID-19 and its policies (social, occupational, economic, physical and mental health)
- COVID-19 information sources
- COVID-19 testing and infection status
- Impacts on schools and schooling
- physical and mental health status
- general health behaviours
- socio-demographics and socio-economic barriers and facilitators of adherence

Most questions are aligned with the constructs in both the COM-B (see **Figure 2**) (12) and Health Belief Models (13, 14). Questions assessing COVID-19 impacts were also chosen to facilitate data harmonisation with international COVID-19 studies involving the NIH and WHO (15). The survey is currently available in **36 languages**, making it legible to the majority of the world's population.

Insert Figure 2 about here

Though the core content of the survey is consistent throughout each release cycle, small modifications have been made as a function of the evolving nature of COVID-19 and public health policies. All surveys are open access and can be found at: https://doi.org/10.17605/OSF.IO/H8RW2. Regardless of the survey content, each questionnaire is designed to take no more than 15-20 minutes to complete.

Global convenience sample: Survey participants are being recruited using online snowball sampling by all global collaborators. The online survey (LimeSurvey©) is distributed through various channels to reach as many people around the world as possible. These channels include professional networks, associations and societies; community organisations; schools and universities; hospitals and health networks; via social media; and personal contacts.

To date, five survey releases have been made (April, May, June, July, and September). Within the current funding that is available, two more releases are planned through January 2021. There are

For peer review only - http://bmjopen.bmj.com/site/about/guidelines.xhtml

several current funding applications which are being evaluated, which if funded, would extend the data collection to eight more release through to January 2022 (see **Figure 3**).

Insert Figure 3 about here

Representative samples in targeted countries: To supplement convenience sampling, we have been conducting parallel national representative sampling in countries where funds are available. Participants in each representative sample are balanced according to age, sex, province/region, education level, and income to ensure representation across these relevant variables. Representative sampling uses polling services to distribute the iCARE survey, generally with internet based sampling methods, though for certain countries, especially low- and middle-income countries (LMICs), there may be a need to conduct telephone and in-person interviews. Representative sampling in targeted countries will ensure global coverage of all geographical locations and socioeconomic gradients. In addition, representative sampling will also allow us to estimate potential biases in the convenience sample data for those countries.

Additional data sources

The Oxford COVID-19 Government Response Tracker (16, 17) systematically collects publicly available information on a variety of indicators of COVID-19 related government policy responses. These policies are then accumulated to provide a variety of indexes as estimates of the total response of an individual country. *Google Mobility Data* (18) provides user mobility trends over time by country and region across different categories of places (e.g., retail, groceries, parks, transit stations, workplaces, and residential), and generates regular “Community Mobility Reports” presented by location. They report the percent change in visits to places like grocery stores and parks within a geographic area. These datasets show how visits and length of stay at different places change compared to baseline. Datasets show trends over several months with the most recent data reflecting the last 2-3 days. *Johns Hopkins Coronavirus Resource Center* (19) has been tracking country-level (and province/state for Canada and the US) case, death and recovery data since the start of the pandemic, and the website is updated multiple times a day. In addition, they provide testing data for US states. The data is drawn from multiple sites.

For peer review only - http://bmjopen.bmj.com/site/about/guidelines.xhtml

Progress to date:

Convenience sampling

Survey 1 of the global convenience sample began on March 27, 2020. When it closed on May 6 we had received surveys from **28,651** people in **137 countries**, including more than 1,000 responses from 4 countries and more than 500 responses from 10 additional countries. Survey 2 of the global convenience sample was launched on May 5, 2020. When it closed on June 8 we had received surveys from **12,576** people in **124** countries, including more than 500 responses from 7 countries. Survey 3 of the global convenience sample was launched on June 8, 2020. When it closed on July 22 we had received surveys from **7,652** people in **100** countries, including more than 500 responses from 3 countries. Survey 4 of the global convenience sample was launched on July 22, 2020. When it closed on September 15, 2020 we had received surveys from **4,102** people in **81** countries, including more than 500 responses from 2 countries.

Representative sampling

To date, six rounds of representative sampling have been captured. Two of these have occurred in Canada (Survey 1: April 9-20, n=3,003 and Survey 3: June 4-17, n=3,005) and Australia (Survey 2: May 1-5, n=1,005 and Survey 3: July 1-7, n=1,051) and one each in the UK (Survey 1: April 3-30, n=2,056) and Ireland (Survey 3: June 22-July 15, 2020, n=1,000). Currently funding will allow us to capture another 2 samples in Canada along with samples from the US, Italy, and Colombia. Additional samples will be captured dependent on funding.

Data harmonization: All data sources will be aggregated at the smallest population level which would ideally be at the level of country, but for those with limited data it might be at the level of continent or for those with large amounts of data it might be at the level of region. Data sources will be tagged based on the date when each participant completed the survey. A series of generalised linear models will be developed to estimate systematic differences in responses between sexes, ethnicities, age-groups, essential worker status, and other key sociodemographic variables. Patterns of missing data will be examined and, where appropriate, accounted for by using multiple imputation techniques (20, 21).

For peer review only - http://bmjopen.bmj.com/site/about/guidelines.xhtml

Statistical analyses: Descriptive analyses, including general linear models or logistic regressions, of the survey data will be provided to explore trends in the main areas represented in the survey. With the magnitude and complexity of the data that is being captured a number of different multilevel modelling techniques will be used. As an example, exploratory iterative generalized least squares (IGLS (22)) models followed by Markov chain Monte Carlo (MCMC) estimation for the final models will likely be used (23). Briefly, this is a Bayesian simulation approach which (after assigning starting values and prior distributions) sequentially samples subsets of parameters from their conditional posterior distributions given current values of the other parameters. This is a very flexible approach used by other groups with comparable data (e.g., NCD-RisC (24)).

For the **representative samples**, appropriate link functions will be tested and used, with the polling company's sampling weights being employed (25-27). All the national representative data will leverage the global data, by pooling all the available information (at any given point in time) and extending our models into a multilevel framework with random effects (intercepts and slopes) at the country levels. By essentially borrowing information from the other countries, this approach will improve the power to obtain robust and precise estimates for any singular country (25, 28).

Ethics and dissemination

Ethics approval

The REB at the co-ordinating study site CIUSSS-NIM provides the primary ethical approval (REB#: 2020-2099 / 03-25-2020). Online consent is provided by participants prior to completing the survey. No personal identifying information is collected from any participant. In addition, several of the collaborating sites have also obtained ethical approval to distribute the survey within their country or institution, though this is not required.

Knowledge translation (KT)

Due to the evolving nature of the COVID-19 pandemic, outputs from analyses will be disseminated in a variety of ways. Regular updates will be posted to the iCARE website (https://mbmc-cmcm.ca/covid19/) and disseminated through the Montreal Behavioural Medicine Centre social media outlets (https://www.facebook.com/CMCMMBMC; https://twitter.com/mbmc_cmcm; https://www.instagram.com/mbmc_cmcm/). Where appropriate press releases and news media will

For peer review only - http://bmjopen.bmj.com/site/about/guidelines.xhtml

be targeted. Of note our study has already received a great deal of media attention, with more than 75 print, radio and television interviews across the globe (as of October 20, 2020; see https://mbmc-cmcm.ca/covid19/media/ for full coverage). Within Canada, we are partnering with the Royal Society of Canada's COVID-19 Task Force to reach the general public, government and national media. Finally, we will also release results through traditional scientific methods, e.g., journal articles and conference presentations. For example, Survey 1 data was presented at the International Behavioural Trials Network Global 2020 Virtual meeting (see https://www.ibtnetwork.org/conference/virtual2020/video-session-2/).

Interpretation

This study will provide high-quality, accelerated and real-time evidence to help us understand the differing impacts of COVID-19 policies, strategies, and communication around the world. It will provide evidence for the effectiveness of evolving country-level policies implemented to reduce the spread of the virus – both in general and among key sub-groups (e.g., younger vs older, ethnic minorities, those with health conditions). The study will also generate evolving evidence to support public health planning, decision-making and responses around the world, including low and middle-income countries.

Limitations: The main limitation of the study is that the survey is being conducted online. Though there is generally good internet access for most high income countries, some LMICs have limited access in certain areas and within certain population sub-groups. This coupled with the convenience sampling method, means that we have the potential for biased samples. Though some of this can be adjusted for based on the representative sampling data, it can't be eliminated completely. Another limitation is the fact that we will be conducting correlation analyses. Though we will be using some sophisticated analytical modelling we can't derive direct causative relationships from the study.

Conclusion: Ultimately, this study will help us understand what public health policies and strategies are working, where, and for whom, which can inform changes (improvements) in policy strategy and communication to help mitigate the spread of COVID-19, especially as countries are now starting to cycle through various waves of the pandemic, and its physical/mental health, social, economic and quality of life impacts**.**

For peer review only - http://bmjopen.bmj.com/site/about/guidelines.xhtml
For peer review only

Data-sharing statement

All completed survey data is anonymous and variables are collected and coded in a way that it would not be possible to identify any specific individual within the survey. Study collaborators are able to obtain access to the data through a standard Research Materials Distribution Agreement (RMDA: see https://mbmc-cmcm.ca/covid19/research/ and http://www.osf.io/nswcm). Sub-analyses of the iCARE data are logged (https://mbmc-cmcm.ca/covid19/apl/) and are openly searchable (https://mbmc-cmcm.ca/covid19/apl/log/).

For peer review only

For peer review only - http://bmjopen.bmj.com/site/about/guidelines.xhtml

References

1. Anderson RM, Heesterbeek H, Klinkenberg D, Hollingsworth TD. How will country-based mitigation measures influence the course of the COVID-19 epidemic? Lancet. 2020;395:931-34.

2. West R, Michie S, Rubin G, Amlot R. Applying principles of behaviour change to reduce SARS-CoV-2 transmission. Nature Human Behaviour. 2020;https://doi.org/10.1038/s41562-020-0887-9.

3. Jarvis CI, Van Zandvoort K, Gimma A, Prem K, Klepac P, Rubin GJ, et al. Quantifying the impact of physical distance measures on the transmission of COVID-19 in the UK. BMC Med. 2020;18(1):124.

4. Davies NG, Kucharski AJ, Eggo RM, Gimma A, Edmunds WJ. Effects of non-pharmaceutical interventions on COVID-19 cases, deaths, and demand for hospital services in the UK: a modelling study. Lancet Public Health. 2020;5(7):e375-e85.

5. Ngonghala CN, Iboi E, Eikenberry S, Scotch M, MacIntyre CR, Bonds MH, et al. Mathematical assessment of the impact of non-pharmaceutical interventions on curtailing the 2019 novel Coronavirus. Math Biosci. 2020;325:108364.

6. Van Bavel J, Baicker K, PS. B, al. E. Using social and behavioural science to support COVID-19 pandemic response. Nature Human Behaviour. 2020.

7. Amárach Research. Impact of Coronavirus - Week 3. 2020;Available at https://amarach.com/news-blog-articles/impact-of-coronavirus-week-3.html: Accessed 27th April 2020.

8. West R, Michie S, Rubin GJ, Amlôt R. Applying principles of behaviour change to reduce SARS-CoV-2 transmission. Nature Human Behaviour. 2020.

9. Milijkovic D. Rational choice and irrational individuals or simply an irrational theory: A critical review of the hypothesis of perfect rationality. Journal of Socio-economics. 2005;34:621-34.

10. Afif Z, Islan W, Calvo-Gonzalez O, Dalton A. Behavioral Science Around the World: Profiles of 10 Countries (English). . eMBeD brief 2018;Washington, D.C.:World Bank Group.

11. Michie S, West R, Campbell R, Brown J, Gainforth H. ABC of Behaviour Change Theories. UK. : Silverback Publishing; 2014.

12. Michie S, van Stralen M, West R. The Behavior Change Wheel: a new method for characterizing and designing behavior change interventions. Implementation Science. 2011;6:42.

13. Rosenstock I, Strecher V, Becker M. Social learning theory and the health belief model. Health Education Quarterly. 1971;15(2):175-83. .

For peer review only

For peer review only - http://bmjopen.bmj.com/site/about/guidelines.xhtml

14. Rosenstock I. The health belief model and preventive health behavior. Health Education Monographs. 1974;2:354-86.

15. National Institutes of Health OoBSSRO. https://www.nlm.nih.gov/dr2/COVID-19_BSSR_Research_Tools.pdf. 2020.

16. Oxford University. http://www.ox.ac.uk/news/2020-03-25-oxford-university-launches-world-s-first-covid-19-government-response-tracker 2020 [

17. Hale T, Webster S, Petherick A, Phillips T, Kira B. Oxford COVID-19 Government Response Tracker, . Blavatnik School of Government 2020.

18. Google. https://www.google.com/covid19/mobility/ 2020 [

19. Johns Hopkins. https://coronavirus.jhu.edu/map.html. 2020.

20. Harrell FE. Regression Modeling Strategies. New York: Springer; 2001.

21. Kleinke K, Reinecke J, Salfrán D, Spiess M. Applied Multiple Imputation. Advantages, Pitfalls, New Developments and Applications in R. New York, NY: Springer; 2020.

22. Goldstein H. Restricted unbiased iterative generalized least-squares estimation. Biometrika. 1989;76(3):622-3.

23. Browne WJ. MCMC Estimation in MLwiN v3.03. University of Bristol: Centre for Multilevel Modelling; 2019.

24. Bixby H, Bentham J, Zhou B, Di Cesare M, Paciorek CJ, Bennett JE, et al. Rising rural body-mass index is the main driver of the global obesity epidemic in adults. Nature. 2019;569(7755):260-4.

25. Valliant R, Dever JA, Kreuter F. Practical Tools for Designing and Weighting Survey Samples. New York, NY: Springer; 2018.

26. Bethlehem J. Weighting. In: Lavrakas PJ, editor. Encyclopedia of Survey Research Methods. Thousand Oaks, California: SAGE Publications, Inc.; 2008. p. 958-60.

27. Heeringa S, West B, Berglund P. Applied Survey Data Analysis. New York: Chapman and Hall/CRC; 2017.

28. Skinner C, Mason B. Weighting in the regression analysis of survey data with a cross-national application. Canadian Journal of Statistics. 2012;40(4):697-711.

For peer review only

For peer review only - http://bmjopen.bmj.com/site/about/guidelines.xhtml

Figure Legends

Figure 1: The theoretical models underpinning the behavioural responses to COVID-19

Figure 2: Measures within Survey 2 mapped onto the COM-B model

Figure 3: Survey release timeline

For peer review only

For peer review only - http://bmjopen.bmj.com/site/about/guidelines.xhtml

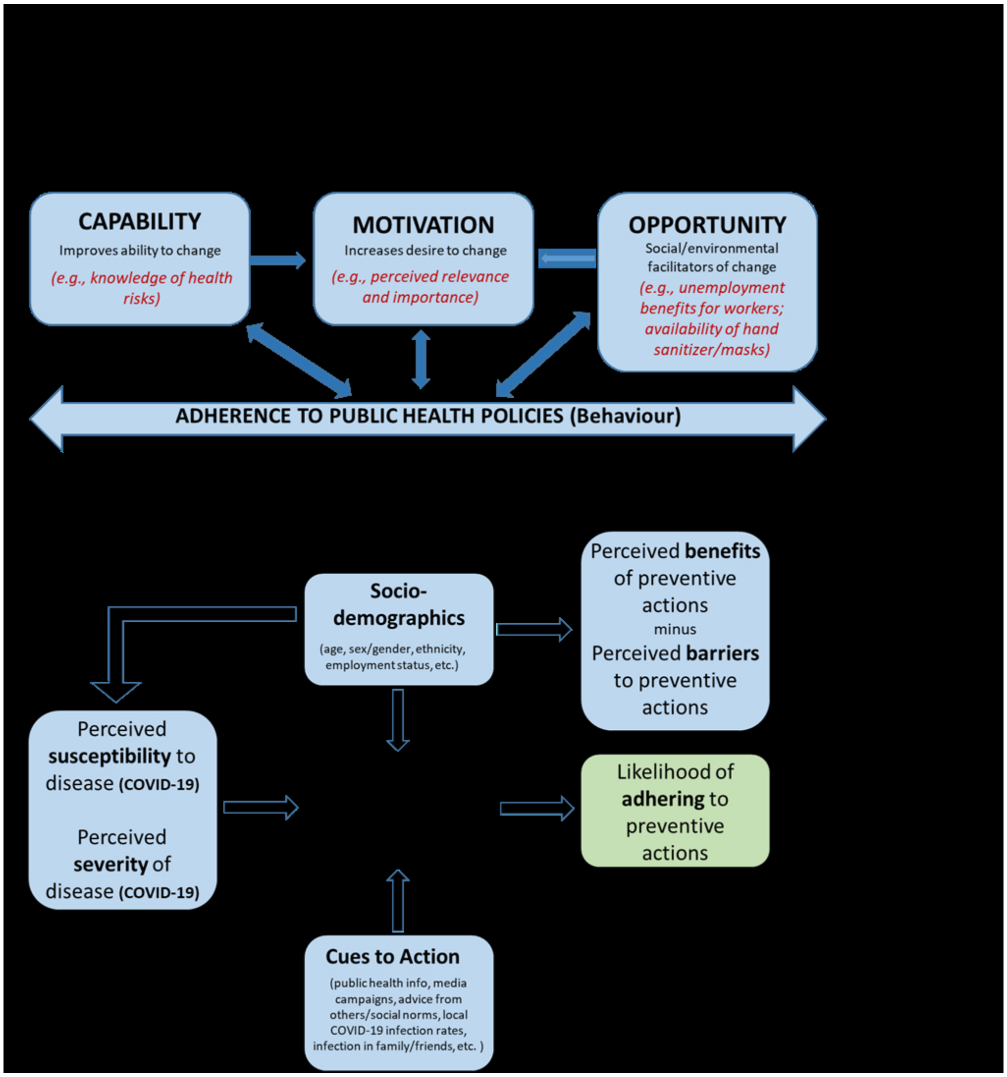

Figure 1
The theoretical models underpinning the behavioural responses to COVID-19

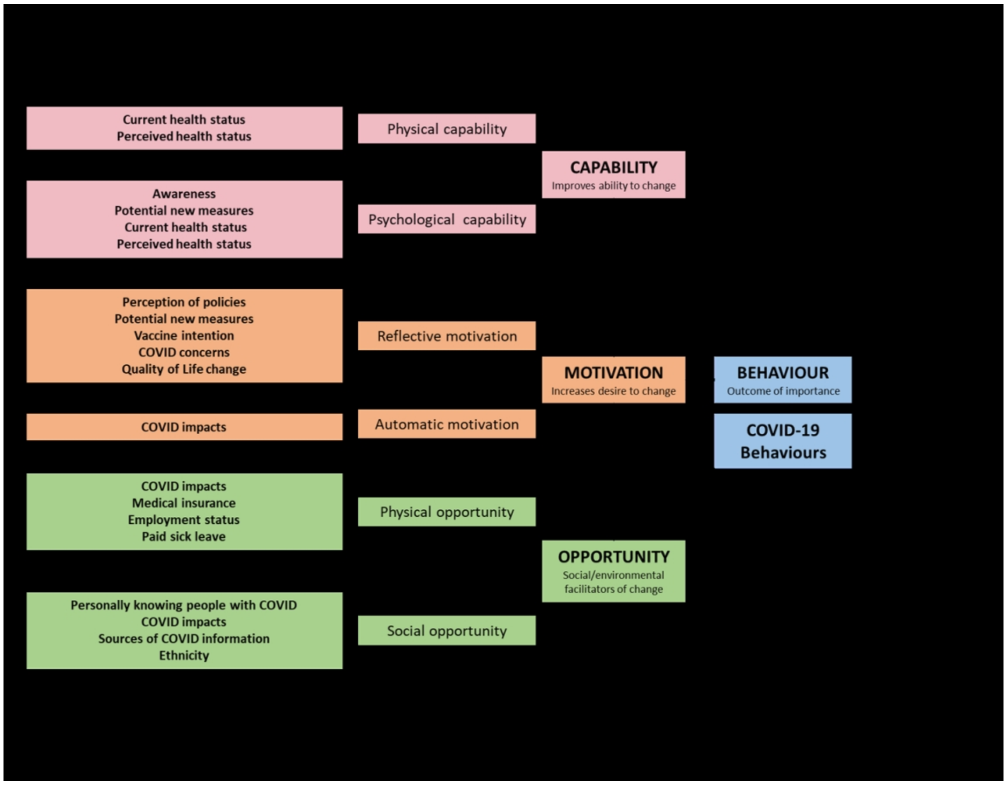

Figure 2
Measures within Survey 2 mapped onto the COM-B model

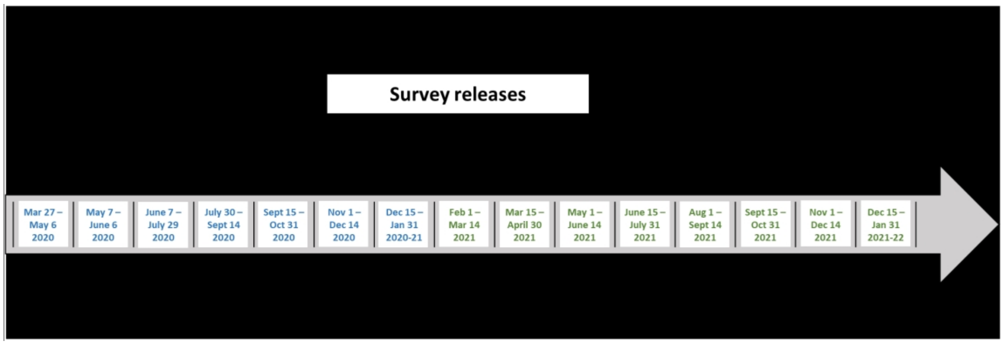

Figure 3
Survey release timeline

Supplementary Material

iCARE Study team

Lead investigators: Kim L. Lavoie, PhD, University of Quebec at Montreal (UQAM) and CIUSSS-NIM, CANADA; Simon L. Bacon, PhD, Concordia University and CIUSSS-NIM, CANADA.

Collaborators (in alphabetical order): ABU DHABI: Zahir Vally, PhD, United Arab Emirates University; AUSTRALIA: Jacqueline Boyle, PhD, Monash University; Joanne Enticott, PhD, Monash University; Shajedur Rahman Shawon, PhD, Centre for Big Data Research in Health, UNSW Medicine; Helena Teede, MD, Monash University; AUSTRIA: Alexandra Kautzky-Willer, MD, Medizinische Universität Wien; BANGLADESH: Arobindu Dash, MS, International University of Business, Agriculture & Technology; BRAZIL: Marilia Estevam Cornelio, PhD, University of Campinas; Marlus Karsten, Universidade do Estado de Santa Catarina - UDESC; Darlan Lauricio Matte, PhD, Universidade do Estado de Santa Catarina - UDESC; CANADA: Ahmed Abou-Setta, PhD, University of Manitoba; Shawn Aaron, PhD, Ottawa Hospital Research Institute; Tracie Barnett, PhD, McGill University; Silvana Barone, MD, University of Montreal; Ariane Belanger-Gravel, PhD, Université Laval; Sarah Bernard, PhD, Université Laval; Lisa Maureen Birch, PhD, Université Laval; Susan Bondy, PhD, University of Toronto - Dalla Lana School of Public Health; Linda Booij, PhD, Concordia University; Roxane Borgès Da Silva, PhD, University of Montreal; Jean Bourbeau, MD, McGill University; Rachel Burns, PhD, Carleton University; Tavis Campbell, PhD, University of Calgary; Linda Carlson, PhD, University of Calgary; Kim Corace, PhD, University of Ottawa; Olivier Drouin, MD, CHU Sainte-Justine/Université de Montréal; Francine Ducharme, MD, University of Montreal; Mohsen Farhadloo, Concordia University; Richard Fleet MD, PhD, Université Laval; Gary Garber, MD, University of Ottawa/Public Health Ontario; Lise Gauvin, PhD, University of Montreal; Jennifer Gordon, PhD, University of Regina; Roland Grad, MD, McGill University; Samir Gupta, MD, University of Toronto; Kim Hellemans, PhD, Carleton University; Catherine Herba PhD, UQAM; Heungsun Hwang, PhD, McGill University; Lisa Kakinami, PhD, Concordia University; Sunmee Kim, PhD, University of Manitoba; Sandra Pelaez, PhD, University of Montreal; Louise Pilote, MD, McGill University; Paul Poirier, MD, Université Laval; Justin Presseau, PhD, University of Ottawa; Eli Puterman, PhD, University of British Columbia; Joshua Rash, PhD, Memorial University; Paula AB Ribeiro, PhD, MBMC; Mohsen Sadatsafavi, PhD, University of British

For peer review only - http://bmjopen.bmj.com/site/about/guidelines.xhtml

Columbia; Paramita Saha Chaudhuri, PhD, McGill University; Jovana Stojanovic, PhD, Concordia University; Eva Suarthana, MD, PhD, University of Montreal / McGill University; Michael Vallis, PhD, Dalhousie University; CHILE: Nicolás Bronfman Caceres, PhD, Universidad Andrés Bello; Manuel Ortiz, PhD, Universidad de La Frontera; Paula Beatriz Repetto, PhD, Universidad Católica de Chile; COLOMBIA: Mariantonia Lemos-Hoyos, PhD, Universidad EAFIT; CYPRUS: Angelos Kassianos, PhD, University of Cyprus; DENMARK: Naja Hulvej Rod, PhD, University of Copenhagen; FRANCE: Mathieu Beraneck, PhD, Université de Paris; CNRS; Greg Ninot, PhD, University of Montpellier; GERMANY: Beate Ditzen, PhD, Heidelberg University; Thomas Kubiak, PhD, Mainz University; GHANA: Sam Codjoe MPhil,MSc, University of Ghana; Lily Kpobi, PhD, University of Ghana; Amos Laar, PhD, University of Ghana; INDIA: Sylvia Fernandez Rao, PhD, Indian Council of Medical Research; Naorem Kiranmala Devi, PhD, University of Delhi; Sanjenbam Meitei, PhD, Manipur University; Suzanne Tanya Nethan, MDS, ICMR-National Institute of Cancer Prevention & Research; Lancelot Pinto, MD, PhD, Hinduja Hospital and Medical Research Centre; Kallur Nava Saraswathy, PhD, University of Delhi; Dheeraj Tumu, MD, World Health Organization (WHO); INDONESIA: Silviana Lestari, MD, PhD, Universitas Indonesia; Grace Wangge, MD, PhD, SEAMEO Regional Center for Food and Nutrition; IRELAND: Molly Byrne, PhD, National University of Ireland, Galway; Jennifer McSharry, PhD, National University of Ireland, Galway; Oonagh Meade, PhD, National University of Ireland, Galway; Gerry Molloy, PhD, National University of Ireland, Galway; Chris Noone, PhD, National University of Ireland, Galway; ISRAEL: Hagai Levine, MD, Hebrew University; Anat Zaidman-Zait, PhD, Tel-Aviv University; ITALY: Stefania Boccia, PhD, Università Cattolica del Sacro Cuore; Ilda Hoxhaj, MD, Università Cattolica del Sacro Cuore, Valeria Raparelli, PhD, Sapienza - University of Rome; Drieda Zaçe, MD, MSc, PhDc, Università Cattolica del Sacro Cuore; JORDAN: Ala'S Aburub, PhD, Isra University; KENYA: Daniel Akunga, PhD, Kenyatta University; Richard Ayah, PhD, University of Nairobi, School Public Health; Chris Barasa, MPH, University of Nairobi, School Public Health; Pamela Miloya Godia, PhD, University of Nairobi; Elizabeth W. Kimani-Murage, PhD, African Population and Health Research Center; Nicholas Mutuku, PhD, University of Kenya; Teresa Mwoma, PhD, Kenyatta University; Violet Naanyu, PhD, Moi University; Jackim Nyamari, PhD, Kenyatta University; Hildah Oburu, PhD, Kenyatta University; Joyce Olenja, PhD, University of Nairobi; Dismas Ongore, PhD, University of Nairobi; Abdhalah Ziraba, PhD, African Population and Health Research Center; LITHUANIA: Emeljanovas Arunas, PhD, Vilnius University; Natalja Fatkulina, PhD, Vilnius University; Brigita Mieziene, PhD, Vilnius University; MALAWI: Chiwoza Bandawe, PhD, University of Malawi; MALAYSIA: Loh Siew Yim,

For peer review only - http://bmjopen.bmj.com/site/about/guidelines.xhtml

For peer review only

PhD, Faculty of medicine, University of Malaya; NEW ZEALAND: Andrea Herbert, PhD, University of Canterbury; Daniela Liggett, PhD, University of Canterbury; Boyd Swinburn, MD, University of Auckland; NIGERIA: Ademola Ajuwon, PhD, University of Ibadan; PAKISTAN: Nisar Ahmed Shar, PhD, CoPI-National Center in Big Data & Cloud Computing; Bilal Ahmed Usmani, PhD, NED University of Engineering and Technology; PERU: Rosario Mercedes Bartolini Martínez, PhD, Instituto de Investigacion Nutricional; Hilary Creed-Kanashiro, M.Phil., Instituto de Investigacion Nutricional; PORTUGAL: Paula Simão, MD, S. Pneumologia de Matosinhos; RWANDA: Pierre Claver Rutayisire, PhD, University Rwanda; SAUDI ARABIA: Abu Zeeshan Bari, PhD, Taibah University; SLOVAKIA: Iveta Nagyova, PhD, PJ Safarik University - UPJS; SOUTH AFRICA: Jason Bantjes, PhD, University of Stellenbosch; Brendon Barnes, PhD, University of Johannesburg; Bronwyne Coetzee, PhD, University of Stellenbosch; Ashraf Khagee, PhD, University of Stellenbosch; Tebogo Mothiba, PhD, University of Limpopo; Rizwana Roomaney, PhD, University of Stellenbosch; Leslie Swartz, PhD University of Stellenbosch; SWEDEN: Anne Berman, PhD, Karolinska Institutet; Nouha Saleh Stattin, MD, Karolinska Institutet; SWITZERLAND: Susanne Fischer, PhD, University of Zurich; TAIWAN: Debbie Hu, MD, MSc, Tainan Municipal Hospital; TURKEY: Yasin Kara, MD, Kanuni Sultan Süleyman Training and Research Hospital, Istanbul; Ceprail Şimşek, MD Health Science University; Bilge Üzmezoğlu, MD, University of Health Science; UGANDA: John Bosco Isunju, PhD, Makerere University School of Public Health; James Mugisha, PhD, University of Uganda; UK: Lucie Byrne-Davis, PhD, University of Manchester; Paula Griffiths, PhD, Loughborough University; Joanne Hart, PhD, University of Manchester; Will Johnson, PhD, Loughborough University; Susan Michie, PhD, University College London; Nicola Paine, PhD, Loughborough University; Emily Petherick, PhD, Loughborough University; Lauren Sherar, PhD, Loughborough University; USA: Robert M. Bilder, PhD, ABPP-CN, University of California, Los Angeles; Matthew Burg, PhD, Yale; Susan Czajkowski, PhD, NIH - National Cancer Institute; Ken Freedland, PhD, Washington University; Sherri Sheinfeld Gorin, PhD, University of Michigan; Alison Holman, PhD, University of California, Irvine; Jiyoung Lee, PhD, University of Alabama; Gilberto Lopez ScD, MA, MPH, Arizona State University and University of Rochester Medical Center; Sylvie Naar, PhD, Florida State University; Michele Okun, PhD, University of Colorado, Colorado Springs; Lynda Powell, PhD, Rush University; Sarah Pressman, PhD, University of California, Irvine; Tracey Revenson, PhD, University of New York City; John Ruiz, PhD, University of Arizona; Sudha Sivaram, PhD, NIH, Center for Global Health; Johannes Thrul, PhD, Johns Hopkins; Claudia Trudel-Fitzgerald, PhD, Harvard T.H. Chan School of Public Health.

For peer review only - http://bmjopen.bmj.com/site/about/guidelines.xhtml

Students (in alphabetical order): AUSTRALIA: Rhea Navani, BSc, Monash University; Kushnan Ranakombu, PhD, Monash University; BRAZIL: Daisuke Hayashi Neto, Unicamp; CANADA: Tair Ben-Porat, PhD, Tel Aviv University; Anda Dragomir, University of Quebec at Montreal (UQAM) and CIUSSS-NIM; Amandine Gagnon-Hébert, BA, UQAM; Claudia Gemme, MSc, UQAM; Vincent Gosselin Boucher, University of Quebec at Montreal (UQAM) and CIUSSS-NIM; Mahrukh Jamil, Concordia University and CIUSSS-NIM; Lisa Maria Käfer, McGill University; Ariany Marques Vieira, MSc, Concordia University; Tasfia Tasbih, Concordia University and CIUSSS-NIM; Maegan Trottier, University of Lethbridge; Robbie Woods, MSc, Concordia University; Reyhaneh Yousefi, Concordia University and CIUSSS-NIM; FRANCE: Tamila Roslyakova, University Montpellier; GERMANY: Lilli Priesterroth, Mainz University; ISRAEL: Shirly Edelstein, Hebrew University-Hadassah School of Public Health; Tanya Goldfrad, Hebrew University-Hadassah School of Public Health; Ruth Snir, Hebrew University-Hadassah School of Public Health; Yifat Uri, Hebrew University-Hadassah School of Public Health; NEW ZEALAND: Mohsen Alyami, University of Auckland; NIGERIA: Comfort Sanuade; SERBIA: Katarina Vojvodic, University of Belgrade.

Community Participants: CANADA: Olivia Crescenzi; Kyle Warkentin; DENMARK: Katya Grinko; INDIA: Lalita Angne; Kulka Bharati, MD; Jigisha Jain; Nikita Mathur, Syncorp Clinical Research; Anagha Mithe; Sarah Nethan, Community Empowerment Lab.

For peer review only

For peer review only - http://bmjopen.bmj.com/site/about/guidelines.xhtml

STANDARD PROTOCOL ITEMS: RECOMMENDATIONS FOR INTERVENTIONAL TRIALS

SPIRIT 2013 Checklist: Recommended items to address in a clinical trial protocol and related documents*

Section/item	ItemNo	Page number	Description
**Administrative information**			
Title	1	1	Descriptive title identifying the study design, population, interventions, and, if applicable, trial acronym
Trial registration	2a	n/a	Trial identifier and registry name. If not yet registered, name of intended registry
	2b	n/a	All items from the World Health Organization Trial Registration Data Set
Protocol version	3	All pages	Date and version identifier
Funding	4	1-2	Sources and types of financial, material, and other support
Roles and responsibilities	5a	1	Names, affiliations, and roles of protocol contributors
	5b	n/a	Name and contact information for the trial sponsor
	5c	2	Role of study sponsor and funders, if any, in study design; collection, management, analysis, and interpretation of data; writing of the report; and the decision to submit the report for publication, including whether they will have ultimate authority over any of these activities
	5d	8	Composition, roles, and responsibilities of the coordinating centre, steering committee, endpoint adjudication committee, data management team, and other individuals or groups overseeing the trial, if applicable (see Item 21a for data monitoring committee)

For peer review only - http://bmjopen.bmj.com/site/about/guidelines.xhtml

**Introduction**			
Background and rationale	6a	6-7	Description of research question and justification for undertaking the trial, including summary of relevant studies (published and unpublished) examining benefits and harms for each intervention
	6b	n/a	Explanation for choice of comparators
Objectives	7	7	Specific objectives or hypotheses
Trial design	8	8	Description of trial design including type of trial (eg, parallel group, crossover, factorial, single group), allocation ratio, and framework (eg, superiority, equivalence, noninferiority, exploratory)
**Methods: Participants, interventions, and outcomes**			
Study setting	9	9	Description of study settings (eg, community clinic, academic hospital) and list of countries where data will be collected. Reference to where list of study sites can be obtained
Eligibility criteria	10	9-10	Inclusion and exclusion criteria for participants. If applicable, eligibility criteria for study centres and individuals who will perform the interventions (eg, surgeons, psychotherapists)
Interventions	11a	n/a	Interventions for each group with sufficient detail to allow replication, including how and when they will be administered
	11b	n/a	Criteria for discontinuing or modifying allocated interventions for a given trial participant (eg, drug dose change in response to harms, participant request, or improving/worsening disease)

For peer review only - http://bmjopen.bmj.com/site/about/guidelines.xhtml

	11c	n/a	Strategies to improve adherence to intervention protocols, and any procedures for monitoring adherence (eg, drug tablet return, laboratory tests)
	11d	n/a	Relevant concomitant care and interventions that are permitted or prohibited during the trial
Outcomes	12	8	Primary, secondary, and other outcomes, including the specific measurement variable (eg, systolic blood pressure), analysis metric (eg, change from baseline, final value, time to event), method of aggregation (eg, median, proportion), and time point for each outcome. Explanation of the clinical relevance of chosen efficacy and harm outcomes is strongly recommended
Participant timeline	13	10	Time schedule of enrolment, interventions (including any run-ins and washouts), assessments, and visits for participants. A schematic diagram is highly recommended (see Figure)
Sample size	14	n/a	Estimated number of participants needed to achieve study objectives and how it was determined, including clinical and statistical assumptions supporting any sample size calculations
Recruitment	15	9-10	Strategies for achieving adequate participant enrolment to reach target sample size
**Methods: Assignment of interventions (for controlled trials)**			
Allocation:			
Sequence generation	16a	n/a	Method of generating the allocation sequence (eg, computer-generated random numbers), and list of any factors for stratification. To reduce predictability of a random sequence, details of any planned restriction (eg, blocking) should be provided in a separate document that is unavailable to those who enrol participants or assign interventions

For peer review only - http://bmjopen.bmj.com/site/about/guidelines.xhtml

Allocation concealment mechanism	16b	n/a	Mechanism of implementing the allocation sequence (eg, central telephone; sequentially numbered, opaque, sealed envelopes), describing any steps to conceal the sequence until interventions are assigned
Implementation	16c	n/a	Who will generate the allocation sequence, who will enrol participants, and who will assign participants to interventions
Blinding (masking)	17a	n/a	Who will be blinded after assignment to interventions (eg, trial participants, care providers, outcome assessors, data analysts), and how
	17b	n/a	If blinded, circumstances under which unblinding is permissible, and procedure for revealing a participant's allocated intervention during the trial
**Methods: Data collection, management, and analysis**			
Data collection methods	18a	9-10	Plans for assessment and collection of outcome, baseline, and other trial data, including any related processes to promote data quality (eg, duplicate measurements, training of assessors) and a description of study instruments (eg, questionnaires, laboratory tests) along with their reliability and validity, if known. Reference to where data collection forms can be found, if not in the protocol
	18b	n/a	Plans to promote participant retention and complete follow-up, including list of any outcome data to be collected for participants who discontinue or deviate from intervention protocols
Data management	19	11	Plans for data entry, coding, security, and storage, including any related processes to promote data quality (eg, double data entry; range checks for data values). Reference to where details of data management procedures can be found, if not in the protocol
Statistical methods	20a	11-12	Statistical methods for analysing primary and secondary outcomes. Reference to where other details of the statistical analysis plan can be found, if not in the protocol

For peer review only - http://bmjopen.bmj.com/site/about/guidelines.xhtml

	20b	11	Methods for any additional analyses (eg, subgroup and adjusted analyses)
	20c	n/a	Definition of analysis population relating to protocol non-adherence (eg, as randomised analysis), and any statistical methods to handle missing data (eg, multiple imputation)
**Methods: Monitoring**			
Data monitoring	21a	n/a	Composition of data monitoring committee (DMC); summary of its role and reporting structure; statement of whether it is independent from the sponsor and competing interests; and reference to where further details about its charter can be found, if not in the protocol. Alternatively, an explanation of why a DMC is not needed
	21b	n/a	Description of any interim analyses and stopping guidelines, including who will have access to these interim results and make the final decision to terminate the trial
Harms	22	n/a	Plans for collecting, assessing, reporting, and managing solicited and spontaneously reported adverse events and other unintended effects of trial interventions or trial conduct
Auditing	23	n/a	Frequency and procedures for auditing trial conduct, if any, and whether the process will be independent from investigators and the sponsor
**Ethics and dissemination**			
Research ethics approval	24	12	Plans for seeking research ethics committee/institutional review board (REC/IRB) approval
Protocol amendments	25	n/a	Plans for communicating important protocol modifications (eg, changes to eligibility criteria, outcomes, analyses) to relevant parties (eg, investigators, REC/IRBs, trial participants, trial registries, journals, regulators)
Consent or assent	26a	12	Who will obtain informed consent or assent from potential trial participants or authorised surrogates, and how (see Item 32)

For peer review only - http://bmjopen.bmj.com/site/about/guidelines.xhtml

	26b	n/a	Additional consent provisions for collection and use of participant data and biological specimens in ancillary studies, if applicable
Confidentiality	27	12	How personal information about potential and enrolled participants will be collected, shared, and maintained in order to protect confidentiality before, during, and after the trial
Declaration of interests	28	2	Financial and other competing interests for principal investigators for the overall trial and each study site
Access to data	29	13-14	Statement of who will have access to the final trial dataset, and disclosure of contractual agreements that limit such access for investigators
Ancillary and post-trial care	30	n/a	Provisions, if any, for ancillary and post-trial care, and for compensation to those who suffer harm from trial participation
Dissemination policy	31a	12	Plans for investigators and sponsor to communicate trial results to participants, healthcare professionals, the public, and other relevant groups (eg, via publication, reporting in results databases, or other data sharing arrangements), including any publication restrictions
	31b	12	Authorship eligibility guidelines and any intended use of professional writers
	31c	12	Plans, if any, for granting public access to the full protocol, participant-level dataset, and statistical code
**Appendices**			
Informed consent materials	32	n/a	Model consent form and other related documentation given to participants and authorised surrogates

For peer review only

Biological specimens	33	n/a	Plans for collection, laboratory evaluation, and storage of biological specimens for genetic or molecular analysis in the current trial and for future use in ancillary studies, if applicable

*It is strongly recommended that this checklist be read in conjunction with the SPIRIT 2013 Explanation & Elaboration for important clarification on the items. Amendments to the protocol should be tracked and dated. The SPIRIT checklist is copyrighted by the SPIRIT Group under the Creative Commons "Attribution-NonCommercial-NoDerivs 3.0 Unported" license.

For peer review only

BMJ Open

An international assessment of the link between COVID-19-related attitudes, concerns and behaviours in relation to public health policies: Optimising policy strategies to improve health, economic and quality of life outcomes (the iCARE Study). Protocol Paper

Journal:	*BMJ Open*
Manuscript ID	bmjopen-2020-046127.R1
Article Type:	Protocol
Date Submitted by the Author:	15-Jan-2021
Complete List of Authors:	Bacon, Simon; Concordia University, Health, Kinesiology, and Applied Physiology; CIUSSS du Nord-de-l'Ile-de-Montreal, Montreal Behavioural Medicine Centre<br>Lavoie, Kim; Université du Québec à Montréal, Psychology; CIUSSS du Nord-de-l'Ile-de-Montreal, Montreal Behavioural Medicine Centre<br>Boyle, Jacqueline; Monash University, Diabetes and Vascular Medicine Unit<br>Stojanovic, Jovana; Concordia University, HKAP; CIUSSS du Nord-de-l'Ile-de-Montreal, Montreal Behavioural Medicine Centre<br>Joyal-Desmarais, Keven; Concordia University, HKAP; CIUSSS du Nord-de-l'Ile-de-Montreal, Montreal Behavioural Medicine Centre
**Primary Subject Heading**:	Public health
Secondary Subject Heading:	Communication, Epidemiology, Health policy
Keywords:	COVID-19, Health policy < HEALTH SERVICES ADMINISTRATION & MANAGEMENT, PREVENTIVE MEDICINE, PUBLIC HEALTH

SCHOLARONE™
Manuscripts

BMJ

I, the Submitting Author has the right to grant and does grant on behalf of all authors of the Work (as defined in the below author licence), an exclusive licence and/or a non-exclusive licence for contributions from authors who are: i) UK Crown employees; ii) where BMJ has agreed a CC-BY licence shall apply, and/or iii) in accordance with the terms applicable for US Federal Government officers or employees acting as part of their official duties; on a worldwide, perpetual, irrevocable, royalty-free basis to BMJ Publishing Group Ltd ("BMJ") its licensees and where the relevant Journal is co-owned by BMJ to the co-owners of the Journal, to publish the Work in this journal and any other BMJ products and to exploit all rights, as set out in our licence.

The Submitting Author accepts and understands that any supply made under these terms is made by BMJ to the Submitting Author unless you are acting as an employee on behalf of your employer or a postgraduate student of an affiliated institution which is paying any applicable article publishing charge ("APC") for Open Access articles. Where the Submitting Author wishes to make the Work available on an Open Access basis (and intends to pay the relevant APC), the terms of reuse of such Open Access shall be governed by a Creative Commons licence – details of these licences and which Creative Commons licence will apply to this Work are set out in our licence referred to above.

Other than as permitted in any relevant BMJ Author's Self Archiving Policies, I confirm this Work has not been accepted for publication elsewhere, is not being considered for publication elsewhere and does not duplicate material already published. I confirm all authors consent to publication of this Work and authorise the granting of this licence.

For peer review only

Title: An international assessment of the link between COVID-19-related attitudes, concerns and behaviours in relation to public health policies: Optimising policy strategies to improve health, economic and quality of life outcomes (the iCARE Study). Protocol Paper.

Brief title: The iCARE study: Protocol paper

Authors: Simon L. Bacon, PhD,[a,b] Kim L. Lavoie, PhD,[a,c] Jacqueline Boyle, MD,[d,e] Jovana Stojanovic, PhD,[a,b] and Keven Joyal-Desmarais, PhD,[a,b] for the iCARE study team*

Word count: 3,356

Author affiliations:

a. Montreal Behavioural Medicine Centre (MBMC), Centre de recherche CIUSSS-NIM, 5400 Gouin West Blvd., Montreal, Quebec, H4J 1C5, Canada

b. Department of Health, Kinesiology, and Applied Physiology, Concordia University, Montreal, Canada

c. Department of Psychology, Université du Québec à Montréal (UQAM), Montreal, Canada

d. Monash Centre for Health Research and Implementation, Monash University, 43-51 Kanooka Grove, Clayton, VIC, 3168 Australia

e. School of Public Health and Preventive Medicine, Monash University, 553 St Kilda Road, Melbourne, VIC, 3004 Australia

* The complete list of iCARE Study collaborators appears in the Supplementary Material.

Contributors statement: All authors contributed to the manuscript including: contributing substantially to conception and design of the study (SLB, KLL, JB, JS, and KJD); drafting the article and revising it critically for important intellectual content (SLB, KLL, JB, JS, and KJD); providing final approval of the version to be published (SLB, KLL, JB, JS, and KJD); and acting as guarantors of the work (SLB, KLL, JB, JS, and KJD).

Version 2: 11th January 2021

For peer review only

Funding statement: The primary source of funding for the iCARE study has been primarily through re-directed funding associated with Montreal Behavioural Medicine Centre, including funds from a Canadian Institutes of Health Research-Strategy for Patient Oriented Research Mentoring Chair (SMC-151518, PI: Dr. Simon L. Bacon), a Fonds de Recherche du Québec: Santé Chair (251618, PI: Dr. Simon L. Bacon), a UQAM Research Chair (1471, PI: Dr. Kim L Lavoie), and Fonds de Recherche du Québec: Santé Senior Research Award (34757, PI: Dr. Kim L Lavoie). The Canadian representative sampling will be funded by the Canadian Institutes of Health Research (MS3-173099, PI: Simon L. Bacon) and the Fonds de Recherche du Québec: Société et Culture (2019-SE1-252541, PI: Dr. Simon L. Bacon). The Australian representative sampling was funded by Monash University and indirectly by the National Health and Medical Research Council and the Medical Research Future Fund (2579, PIs: Drs. Helena Teede and Jacqueline Boyle). The Irish representative sampling was funded by the Health Research Board and the Irish Research Council (COV19-2020-097, PI: Dr. Gerard J. Molloy). The UK representative sampling was funded by CALIBRE research funding, provided by Loughborough University, UK (5705, PI: Dr. Nicola J. Paine). None of the funders were involved in the study design.

Study registration: N/A

Competing interests:

Dr. Bacon has received consultancy fees from Merck for the development of behavior change continuing education modules, speaker fees from Novartis and Janssen, and has served on advisory boards for Bayer, Sanofi, and Sojecci Inc, none of which are related to the current article.

Dr. Lavoie has served on the advisory board for Schering-Plough, Takeda, AbbVie, Almirall, Janssen, GSK, Boehringer Ingelheim (BI), and Sojecci Inc, and has received sponsorship for investigator-generated research grants from GlaxoSmithKline (GSK) and AbbVie, speaker fees from GSK, Astra-Zeneca, Astellas, Novartis, Takeda, AbbVie, Merck, Boehringer Ingelheim, Bayer, Pfizer and Air Liquide, and support for educational materials from Merck, none of which are related to the current article.

Drs. Boyle, Stojanovic, and Joyal-Desmarais have no competing interests to declare.

For peer review only

For peer review only - http://bmjopen.bmj.com/site/about/guidelines.xhtml

Address for correspondence:

Simon L. Bacon, PhD, FTOS, FCCS, FABMR

Montreal Behavioral Medicine Centre

CIUSSS-NIM

Email: simon.bacon@concordia.ca

Twitter: @sbacon20, @mbmc_cmcm

For peer review only

For peer review only - http://bmjopen.bmj.com/site/about/guidelines.xhtml

Abstract:

Introduction: In the context of a highly contagious virus with only recently approved vaccines and no cure, the key to slowing the spread of the COVID-19 disease and successfully transitioning through the phases of the pandemic, including vaccine uptake, is public adherence to rapidly evolving behaviour-based public health policies. The overall objective of the iCARE Study is to assess public awareness, attitudes, concerns, and behavioural responses to COVID-19 public health policies, and their impacts, on people around the world, and to link behavioural survey data with policy, mobility, and case data to provide behavioural science, data-driven recommendations to governments on how to optimise current policy strategies to reduce the impact of the COVID-19 pandemic.

Methods and analyses: The iCARE study (www.mbmc-cmcm.ca/covid19) utilises a multiple cross-sectional survey design to capture self-reported information on a variety of COVID-19 related variables from individuals around the globe. Survey data is captured using two data capture methods, convenience and representative sampling. This data is then linked to open access data for policies, cases, and population movement.

Ethics and Dissemination: The primary ethical approval was obtained from the co-ordinating site, the CIUSSS-NIM (REB#: 2020-2099 / 03-25-2020). This study will provide high-quality, accelerated and real-time evidence to help us understand the effectiveness of evolving country-level policies and communication strategies to reduce the spread of the COVID-19. Due to the urgency of the pandemic, results will be disseminated in a variety of ways, including policy briefs, social media posts, press releases, and through regular scientific methods.

Registration: N/A

Keywords: Evidence-based policies; Behaviour change; COVID-19

For peer review only

For peer review only - http://bmjopen.bmj.com/site/about/guidelines.xhtml

Strengths and limitations of this study

- This is a large, international study that has data captured from over 150 countries.
- The survey was constructed around well recognised behavioural theories and frameworks.
- The study is primarily being conducted online which may limit some of the generalisability of the data that is available, especially in lower and middle income countries.
- The primary data capture method is through snowball sampling, which is likely to create some bias in the sample. However, some of this can be adjusted using weightings from the representative samples that are being collected.
- A key strength of the study is that it has been developed to provide constructive policy and communication strategy data which can be implemented by governments to improve adherence to COVID-19 mitigation methods.

For peer review only

For peer review only - http://bmjopen.bmj.com/site/about/guidelines.xhtml

Abbreviations:

CIUSSS-NIM – Centre intégré universitaire de santé et de services sociaux du Nord-de-l'Île-de-Montréal

COM-B – Capability, Opportunity, Motivation-Behaviour Model

iCARE – International assessment of the link between COVID-19-related attitudes, concerns and behaviours in relation to public health policies

IGLS – Iterative generalized least squares

LMIC – Low- and Middle-income countries

MBMC – Montreal Behavioural Medicine Centre

MCMC – Markov chain Monte Carlo

OxCGRT – Oxford COVID-19 Government Response Tracker

UQAM – Université du Québec à Montréal

For peer review only

For peer review only - http://bmjopen.bmj.com/site/about/guidelines.xhtml

Introduction:

With only recently approved vaccines and no cure, the key to slowing the spread of COVID-19 and successfully transitioning through the phases of the pandemic, is ***public adherence*** to unprecedented and rapidly evolving **behaviour-based** public health policies (1, 2). To date, adherence to these policies has been critical to reducing the spread of COVID-19 and have ranged from personal hygiene measures (e.g., hand washing) to strict lockdown measures (e.g., business and school closures) (3-5). However, adherence to most of these policies requires making behavioural changes that may come with significant personal, social and economic costs, which may undermine their impact (6). For example, despite public health messages promoting the 'advantages' of adhering to COVID-19 mitigation measures, adherence to policies that may come with high personal costs (i.e., physical distancing) have been much poorer (54%) than for other 'less costly' behaviours like hand washing (90%) (7). Further, as we look towards changing lockdown measures, people's willingness to adhere to evolving government recommendations (e.g., school and store reopening's, receiving vaccines) will also be critical for re-engaging the economy whilst minimising the potential for future waves of the pandemic. Unfortunately, policy variations between and within countries, have created public confusion and uncertainty about government policy motives (8). In addition, governments have predominantly designed policies based on how they believe people 'should' behave and have ascribed little consideration to what we know about how people actually behave (9, 10).

Decades of behavioural science research has revealed that human behaviour is predictable and modifiable (11). Multiple factors are likely to predict why people adhere (or not) to various public health measures, which, in the context of COVID-19, can be defined using two related behaviour prediction models: 1) *The Capability, Opportunity, Motivation-Behaviour (COM-B) Model* (2, 12), which predicts that behaviour change depends on: awareness of prevention measures and the ability to enact them (capability), the belief that measures are personally relevant and important (motivation), and having the social and environmental resources required to adopt the behaviour (opportunity) (see **Figure 1a**); and 2) *The Health Beliefs Model* (13, 14), which posits that in adopting disease prevention measures, a person's belief in the personal threat(s) posed by the disease, together with a person's belief in the importance and effectiveness of recommended behaviours, will predict the likelihood a person adopting (or not) a particular behaviour (**Figure 1b**). In the context of this unprecedented health, social, and economic crisis, where the global need for adherence to

For peer review only - http://bmjopen.bmj.com/site/about/guidelines.xhtml

rapidly evolving public health policies has never been greater, our understanding of the determinants of adherence at each phase of the pandemic, and as a function of various policies, is critical for effective policy planning, communication, and effectiveness.

Insert Figure 1 about here

The overall goal of the iCARE Study is to assess public awareness, attitudes, concerns, and behavioural responses to COVID-19 public health policies, and their impacts, on people around the world (www.mbmc-cmcm.ca/covid19), and to link behavioural survey data with policy, mobility, and case data to provide behavioural science, data-driven recommendations to governments on how to optimise current policy strategies to reduce the impact of the COVID-19 pandemic worldwide. Specifically, we will address the following:

1) What are the **key individual characteristics** (e.g., sociodemographic; psychological; behavioural; physical/mental health; and economic) that **are associated with adherence** to major COVID-19 public health policies *in general* and by *country*?
2) To what extent are COVID-19 **attitudes, beliefs and concerns associated with adherence**, and how does this vary across key subgroups (e.g., age, sex, income, family/household structure, ethnic groups, those with health conditions, etc.)?
3) What are the short- and medium-term **impacts of COVID-19 and its public health policies**, and how do they vary as a function of key individual characteristics in *general* and by *country*?
4) Which policies and strategies are associated with better (and worse) adherence, are most (and least) effective at reducing infection rates, and positively impact economic growth (where appropriate)? As well as, identifying in whom these polices and strategies worked (and did not work).
5) The development of **behavioural science, data-driven, tailored recommendations,** that governments could use to optimise policy and communication strategies to improve adherence, as well as, health, economic, and quality of life outcomes.

For peer review only - http://bmjopen.bmj.com/site/about/guidelines.xhtml
For peer review only

Methods and analysis

Study design:

The iCARE Study is a Canadian-led, ongoing, multi-wave international study involving the collaboration of more than **190 international researchers** from over **40 countries** (see Supplementary Material). It utilises a multiple cross-sectional survey design (each approximately 6 weeks apart) to capture self-reported information on a variety of COVID-19 related variables from individuals around the globe. Survey data is captured using two data capture methods, convenience and representative sampling (see details below). This data is then coupled to open access data for policies, cases, and population movement. The study is managed by the Montreal Behavioural Medicine Centre (MBMC: a joint Centre intégré universitaire de santé et de services sociaux du Nord-de-l'Île-de-Montréal (CIUSSS-NIM) / Université du Québec à Montréal (UQAM) / Concordia University academic research and training centre).

Patient and Public Involvement (PPI) Statement

Given the significance and broad impact of the COVID-19 pandemic PPI is crucial for effective research in this area. More importantly, given the global nature of the iCARE study it has been critical to have individuals from multiple settings included in the development of the various elements and items in the survey. To this end, we consulted with over 190 multidisciplinary collaborators (including experts from the behavioural sciences, medicine and infectious disease, public health, epidemiology, statistics, and implementation science) from more than 40 countries including researchers, clinicians, students, and members of the general public in the development and design of the iCARE study (see Supplementary Material for the iCARE team). In addition, throughout our data analysis process we have engaged critical end users, including government officials, the public, the news media, in defining areas that need critical input for which the iCARE study is able to address.

The iCARE survey:

The core elements of the survey assess the following domains:

- awareness of local COVID-19 public health policies
- attitudes/beliefs about local COVID-19 policies
- behavioural responses to local COVID-19 policies

For peer review only - http://bmjopen.bmj.com/site/about/guidelines.xhtml

- perceived concerns about COVID-19
- the impacts of COVID-19 and its policies (social, occupational, economic, quality of life, physical and mental health)
- COVID-19 information sources
- COVID-19 testing and infection status
- impacts on schools and education
- physical and mental health status
- general health behaviours, including vaccine history, attitudes, and behaviours
- socio-demographics and socio-economic barriers and facilitators of adherence

Most questions are aligned with the constructs in both the COM-B (see **Figure 2**) (12) and Health Belief Models (13, 14). Questions assessing COVID-19 impacts were also chosen to facilitate data harmonisation with international COVID-19 studies involving the NIH and WHO (15). The survey is currently available in **36 languages**, making it legible to the majority of the world's population.

Insert Figure 2 about here

Though the core content of the survey is consistent throughout each release cycle, small modifications have been made as a function of the evolving nature of COVID-19 and public health policies. All surveys are open access and can be found at: https://osf.io/nswcm. Regardless of the survey content, each questionnaire is designed to take no more than 15-20 minutes to complete.

Global convenience sample: Survey participants are being recruited using online snowball sampling by all global collaborators. The online survey (LimeSurvey©) is distributed through various channels to reach as many people around the world as possible. These channels include professional networks, associations and societies; community organisations; schools and universities; hospitals and health networks; via social media; and personal contacts. The central study coordination group creates a variety of email, social media, and public facing materials for each survey round which are then translated and provided to each collaborator. There are also a series of instructional tools which

For peer review only - http://bmjopen.bmj.com/site/about/guidelines.xhtml

collaborators can use that provide information and examples of ways in which they can distribute the survey through their local country networks.

To date, there have been seven survey releases (April, May, June, July, September, November, and December). There are several current funding applications that are being reviewed, which if funded, would extend the data collection to eight more releases through to January 2022 (see **Figure 3**).

Insert Figure 3 about here

Representative samples in target countries: To supplement convenience sampling, we have been conducting parallel national representative sampling in countries where funds are available. Participants in each representative sample are balanced according to age, sex, province/region, education level, and income to ensure representation across these relevant variables. Representative sampling uses polling services to distribute the iCARE survey, generally with internet based sampling methods, though for certain countries, especially low- and middle-income countries (LMICs), there may be a need to conduct telephone and in-person interviews. For example in Canada, we have used Leger © polling services, who recruit participants aged 18 and over through their Léo online panel (LégerWeb.com). This panel includes over 400,000 Canadians, most of whom (60%) have been recruited within the past 10 years. Two thirds of the panel were recruited randomly by telephone, with the remainder recruited via publicity and social media. Using data from Statistics Canada, results are weighted within each province according to the sex and age of the respondents in order to make their profiles representative of the actual population within each Canadian province. Then, the weight of each province is adjusted to make it representative of their actual weight within the Canadian federation. Representative sampling in targeted countries will enable global coverage of most geographical locations and socioeconomic gradients. In addition, representative sampling will also allow us to estimate potential biases in the convenience sample data for those countries.

Additional data sources

The Oxford COVID-19 Government Response Tracker (16, 17) systematically collects publicly available information on a variety of indicators of COVID-19 related government policy responses. These policies are then accumulated to provide a variety of indexes as estimates of the total response of an

For peer review only - http://bmjopen.bmj.com/site/about/guidelines.xhtml

individual country. *Google Mobility Data* (18) provides user mobility trends over time by country and region across different categories of places (e.g., retail, groceries, parks, transit stations, workplaces, and residential), and generates regular "Community Mobility Reports" presented by location. They report the percent change in visits to places like grocery stores and parks within a geographic area. These datasets show how visits and length of stay at different places change compared to baseline. Datasets show trends over several months with the most recent data reflecting the last 2-3 days. *Johns Hopkins Coronavirus Resource Center* (19, 20) has been tracking country-level (and province/state for Canada and the US) case, death and recovery data since the start of the pandemic, and the website is updated multiple times a day. In addition, they provide testing data for US states. The data is drawn from multiple sites.

Progress to date:

Convenience sampling

Survey 1 of the global convenience sample began on March 27, 2020. When it closed on May 6 we had received surveys from **28,651** people in **137 countries**, including more than 1,000 responses from 4 countries and more than 500 responses from 10 additional countries. Survey 2 of the global convenience sample was launched on May 5, 2020. When it closed on June 8 we had received surveys from **12,576** people in **124** countries, including more than 500 responses from 7 countries. Survey 3 of the global convenience sample was launched on June 8, 2020. When it closed on July 22 we had received surveys from **7,652** people in **100** countries, including more than 500 responses from 3 countries. Survey 4 of the global convenience sample was launched on July 22, 2020. When it closed on September 15, 2020 we had received surveys from **4,102** people in **81** countries, including more than 500 responses from 2 countries. Survey 5 of the global convenience sample was launched on September 15, 2020. When it closed on November 3, 2020 we had received surveys from **3,404** people in **87** countries, including more than 500 responses from 2 countries. Survey 6 of the global convenience sample was launched on November 3, 2020. When it closed on December 15, 2020 we had received surveys from **2,451** people in **73** countries, including more than 500 responses from 1 country.

Representative sampling

For peer review only - http://bmjopen.bmj.com/site/about/guidelines.xhtml

To date, seven rounds of representative sampling have been captured. Three of these have occurred in Canada (Survey 1: April 9-20, n=3,003, Survey 3: June 4-17, n=3,005, and Survey 6: October 28-November 10, n=3,005), two in Australia (Survey 2: May 1-5, n=1,005 and Survey 3: July 1-7, n=1,051) and one each in the UK (Survey 1: April 3-30, n=2,056) and Ireland (Survey 3: June 22-July 15, 2020, n=1,000). Current funding will allow us to capture another 2 samples in Canada along with samples from the US, Italy, and Colombia. Additional samples will be captured dependent on funding.

Data harmonization: Initially, all data sources will be aggregated at the country level, as a function of available data. However, for those with limited data it might be at the level of continent and for those with large amounts of data we may also be able to provide data at the level of region. Data sources will be tagged based on the date when each participant completed the survey. A series of generalised linear models will be developed to estimate systematic differences in responses between sexes, ethnicities, age-groups, essential worker status, and other key sociodemographic variables. Patterns of missing data will be examined and, where appropriate, accounted for by using multiple imputation techniques (21, 22). In countries where there is sufficient data in the convenience sample, we will apply weights to allow the data to provide national approximations (23-25).

Statistical analyses: Descriptive analyses, including general linear models or logistic regressions, of the survey data will be provided to explore trends in the main areas represented in the survey. Where possible, the psychometric properties of the various elements of the survey will be explored. This will also include a variety of clustering techniques, e.g., principal components analyses (PCA) or factor analyses, to create appropriate sub-scales. For instance, to cluster and reduce the dimensionality of the COVID-19 impact questions for Surveys 2 to 4, we performed a PCA on the polychoric correlation matrix of the COVID-19 impacts variables. We used an orthogonal (varimax) rotation in order to distribute the component loadings. We identified different impact components based on the Kaiser criterion (eigenvalue >1.0) (26), scree plot, component loadings (> 0.4) and components interpretability. For the main study questions (see above), with the magnitude and complexity of the data that is being captured a number of different multilevel modelling techniques will be used. As an example, exploratory iterative generalized least squares (IGLS (27)) models followed by Markov chain Monte Carlo (MCMC) estimation for some models will likely be used (28). Briefly, this is a Bayesian simulation approach which (after assigning starting values and prior distributions) sequentially

For peer review only - http://bmjopen.bmj.com/site/about/guidelines.xhtml

samples subsets of parameters from their conditional posterior distributions given current values of the other parameters. This is a very flexible approach used by other groups with comparable data (e.g., NCD-RisC (29)). For instance, using this approach we are evaluating how the perception of government recommendations and the population's behaviour regarding facemasks wearing varies according to the date of policy implementation in five targeted countries (Canada, USA, Colombia, Brazil and France) and how this then tracks onto case rates.

For the **representative samples**, appropriate link functions will be tested and used, with the polling company's sampling weights being employed (23-25). All the national representative data will leverage the global data, by pooling all the available information (at any given point in time) and extending our models into a multilevel framework with random effects (intercepts and slopes) at the country levels. By essentially borrowing information from the other countries, this approach will improve the power to obtain robust and precise estimates for any singular country (23, 30). In addition, where possible, we will leverage the representative samples to be able to validate the 'representativeness' of the data captured in the global sample. These analyses may provide insights into potential areas of bias and so that appropriate weightings that can be applied to the global sample.

Ethics and dissemination

Ethics approval

The REB at the co-ordinating study site CIUSSS-NIM provides the primary ethical approval (REB#: 2020-2099 / 03-25-2020). Online consent is provided by participants prior to completing the survey. No personal identifying information is collected from any participant. In addition, several of the collaborating sites have also obtained ethical approval to distribute the survey within their country or institution, though this is not required.

Knowledge translation (KT)

Due to the evolving nature of the COVID-19 pandemic, outputs from analyses will be disseminated in a variety of ways. Regular updates will be posted to the iCARE website (https://mbmc-cmcm.ca/covid19/) and disseminated through the Montreal Behavioural Medicine Centre social media outlets (https://www.facebook.com/CMCMMBMC; https://twitter.com/mbmc_cmcm;

For peer review only - http://bmjopen.bmj.com/site/about/guidelines.xhtml

https://www.instagram.com/mbmc_cmcm/). Where appropriate press releases and news media will be targeted. Of note our study has already received a great deal of media attention, with more than 75 print, radio and television interviews across the globe (as of October 20, 2020; see https://mbmc-cmcm.ca/covid19/media/ for full coverage). Within Canada, we are partnering with the Royal Society of Canada's COVID-19 Task Force to reach the general public, government and national media. Finally, we will also release results through traditional scientific methods, e.g., journal articles and conference presentations. For example, Survey 1 data was presented at the International Behavioural Trials Network Global 2020 Virtual meeting (see https://www.ibtnetwork.org/conference/virtual2020/video-session-2/).

Interpretation

This study will provide high-quality, accelerated and real-time evidence to help us understand the differing impacts of COVID-19 policies, strategies, and communication around the world. It will provide evidence for the effectiveness of evolving policies implemented to reduce the spread of the virus – both in general and among key sub-groups (e.g., younger vs older, ethnic minorities, those with health conditions). The study will also generate evolving evidence to support public health planning, decision-making and responses around the world, including low and middle-income countries. Examples of the results to date can be found at https://mbmc-cmcm.ca/covid19/research/stats/ and https://mbmc-cmcm.ca/covid19/research/infog/. Of note, the iCARE study has provided data to the Canadian (Federal), Irish, Province of Ontario (Canada), and State of Victoria (Australia) governments, covering polices ranging from facemasks, contact tracing applications, and COVID-19 vaccine uptake.

Limitations: The main limitation of the study is that the survey is being conducted online. Though there is generally good internet access for most high income countries and even some LMIC's (e.g., India), some LMICs have limited access in certain areas and within certain population sub-groups. This coupled with the convenience sampling method, means that there may be some degree of sample bias. Though some of this can be adjusted for based on the representative sampling data, it can't be eliminated completely. Moreover, the fact that the iCARE survey is available in 36 languages means that certain marginalized groups (e.g., immigrants to certain countries, like Canada, the US and France, which are highly represented) will likely be able to complete the survey in their native

For peer review only - http://bmjopen.bmj.com/site/about/guidelines.xhtml

language. This may help increase participation among those who might otherwise be excluded due to language barriers. Another limitation is the fact that we will be conducting correlation analyses. Though we will be using some sophisticated analytical modelling we can't derive direct causative relationships from the study. However, our main interest is in temporal changes in attitudes and behaviours as the pandemic evolves, so analysing repeated cross sectional cohorts still allows us to meet our study objectives.

Conclusion: Ultimately, this study will help us understand what public health policies and strategies are working, where, and for whom, which can inform changes (improvements) in policy strategy and communication to help mitigate the spread of COVID-19, especially as countries are now starting to cycle through various waves of the pandemic, and its physical/mental health, social, economic and quality of life impacts**.**

Data-sharing statement

All completed survey data is anonymous and variables are collected and coded in a way that it would not be possible to identify any specific individual within the survey. Study collaborators are able to obtain access to the data through a standard Research Materials Distribution Agreement (RMDA: see https://mbmc-cmcm.ca/covid19/research/ and http://www.osf.io/nswcm). Sub-analyses of the iCARE data are logged (https://mbmc-cmcm.ca/covid19/apl/) and are openly searchable (https://mbmc-cmcm.ca/covid19/apl/log/).

For peer review only - http://bmjopen.bmj.com/site/about/guidelines.xhtml

References

1. Anderson RM, Heesterbeek H, Klinkenberg D, Hollingsworth TD. How will country-based mitigation measures influence the course of the COVID-19 epidemic? Lancet. 2020;395:931-34.

2. West R, Michie S, Rubin G, Amlot R. Applying principles of behaviour change to reduce SARS-CoV-2 transmission. Nature Human Behaviour. 2020;https://doi.org/10.1038/s41562-020-0887-9.

3. Jarvis CI, Van Zandvoort K, Gimma A, Prem K, Klepac P, Rubin GJ, et al. Quantifying the impact of physical distance measures on the transmission of COVID-19 in the UK. BMC Med. 2020;18(1):124.

4. Davies NG, Kucharski AJ, Eggo RM, Gimma A, Edmunds WJ. Effects of non-pharmaceutical interventions on COVID-19 cases, deaths, and demand for hospital services in the UK: a modelling study. Lancet Public Health. 2020;5(7):e375-e85.

5. Ngonghala CN, Iboi E, Eikenberry S, Scotch M, MacIntyre CR, Bonds MH, et al. Mathematical assessment of the impact of non-pharmaceutical interventions on curtailing the 2019 novel Coronavirus. Math Biosci. 2020;325:108364.

6. Van Bavel J, Baicker K, PS. B, al. E. Using social and behavioural science to support COVID-19 pandemic response. Nature Human Behaviour. 2020.

7. Amárach Research. Impact of Coronavirus - Week 3. 2020;Available at https://amarach.com/news-blog-articles/impact-of-coronavirus-week-3.html: Accessed 27th April 2020.

8. West R, Michie S, Rubin GJ, Amlôt R. Applying principles of behaviour change to reduce SARS-CoV-2 transmission. Nature Human Behaviour. 2020.

9. Milijkovic D. Rational choice and irrational individuals or simply an irrational theory: A critical review of the hypothesis of perfect rationality. Journal of Socio-economics. 2005;34:621-34.

10. Afif Z, Islan W, Calvo-Gonzalez O, Dalton A. Behavioral Science Around the World: Profiles of 10 Countries (English). . eMBeD brief 2018;Washington, D.C.:World Bank Group.

11. Michie S, West R, Campbell R, Brown J, Gainforth H. ABC of Behaviour Change Theories. UK. : Silverback Publishing; 2014.

12. Michie S, van Stralen M, West R. The Behavior Change Wheel: a new method for characterizing and designing behavior change interventions. Implementation Science. 2011;6:42.

13. Rosenstock I, Strecher V, Becker M. Social learning theory and the health belief model. Health Education Quarterly. 1971;15(2):175-83. .

For peer review only - http://bmjopen.bmj.com/site/about/guidelines.xhtml

14. Rosenstock I. The health belief model and preventive health behavior. Health Education Monographs. 1974;2:354-86.

15. National Institutes of Health OoBSSRO. https://www.nlm.nih.gov/dr2/COVID-19_BSSR_Research_Tools.pdf. 2020.

16. Oxford University. http://www.ox.ac.uk/news/2020-03-25-oxford-university-launches-world-s-first-covid-19-government-response-tracker 2020 [

17. Hale T, Webster S, Petherick A, Phillips T, Kira B. Oxford COVID-19 Government Response Tracker, . Blavatnik School of Government 2020.

18. Google. https://www.google.com/covid19/mobility/ 2020 [

19. Dong E, Du H, Gardner L. An interactive web-based dashboard to track COVID-19 in real time. The Lancet Infectious Diseases. 2020;20(5):533-4.

20. Johns Hopkins. https://coronavirus.jhu.edu/map.html. 2020.

21. Harrell FE. Regression Modeling Strategies. New York: Springer; 2001.

22. Kleinke K, Reinecke J, Salfrán D, Spiess M. Applied Multiple Imputation. Advantages, Pitfalls, New Developments and Applications in R. New York, NY: Springer; 2020.

23. Valliant R, Dever JA, Kreuter F. Practical Tools for Designing and Weighting Survey Samples. New York, NY: Springer; 2018.

24. Bethlehem J. Weighting. In: Lavrakas PJ, editor. Encyclopedia of Survey Research Methods. Thousand Oaks, California: SAGE Publications, Inc.; 2008. p. 958-60.

25. Heeringa S, West B, Berglund P. Applied Survey Data Analysis. New York: Chapman and Hall/CRC; 2017.

26. Kaiser HF. The Application of Electronic Computers to Factor Analysis. Educational and Psychological Measurement. 1960;20(1):141-51.

27. Goldstein H. Restricted unbiased iterative generalized least-squares estimation. Biometrika. 1989;76(3):622-3.

28. Browne WJ. MCMC Estimation in MLwiN v3.03. University of Bristol: Centre for Multilevel Modelling; 2019.

29. Bixby H, Bentham J, Zhou B, Di Cesare M, Paciorek CJ, Bennett JE, et al. Rising rural body-mass index is the main driver of the global obesity epidemic in adults. Nature. 2019;569(7755):260-4.

30. Skinner C, Mason B. Weighting in the regression analysis of survey data with a cross-national application. Canadian Journal of Statistics. 2012;40(4):697-711.

For peer review only - http://bmjopen.bmj.com/site/about/guidelines.xhtml

For peer review only

For peer review only - http://bmjopen.bmj.com/site/about/guidelines.xhtml

Figure Legends

Figure 1: The theoretical models underpinning the behavioural responses to COVID-19

Figure 2: Measures within Survey 2 mapped onto the COM-B model

Figure 3: Survey release timeline

For peer review only

For peer review only - http://bmjopen.bmj.com/site/about/guidelines.xhtml

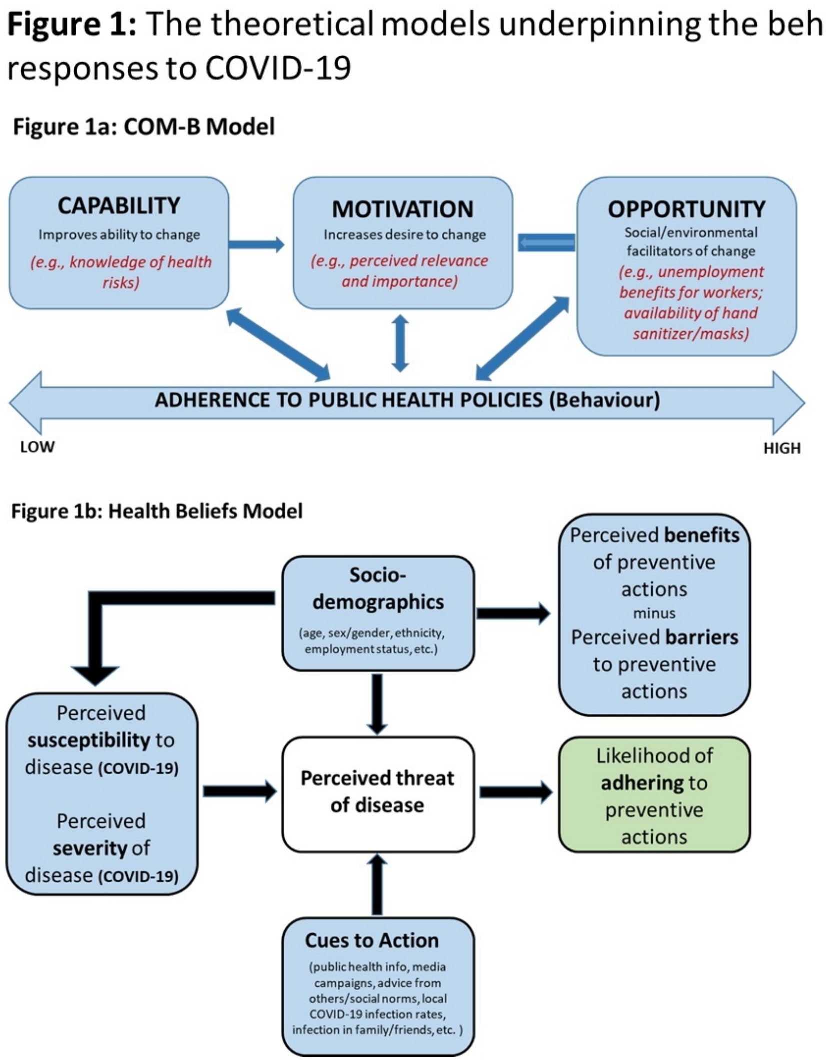

Figure 1: The theoretical models underpinning the behavioural responses to COVID-19

269x287mm (96 x 96 DPI)

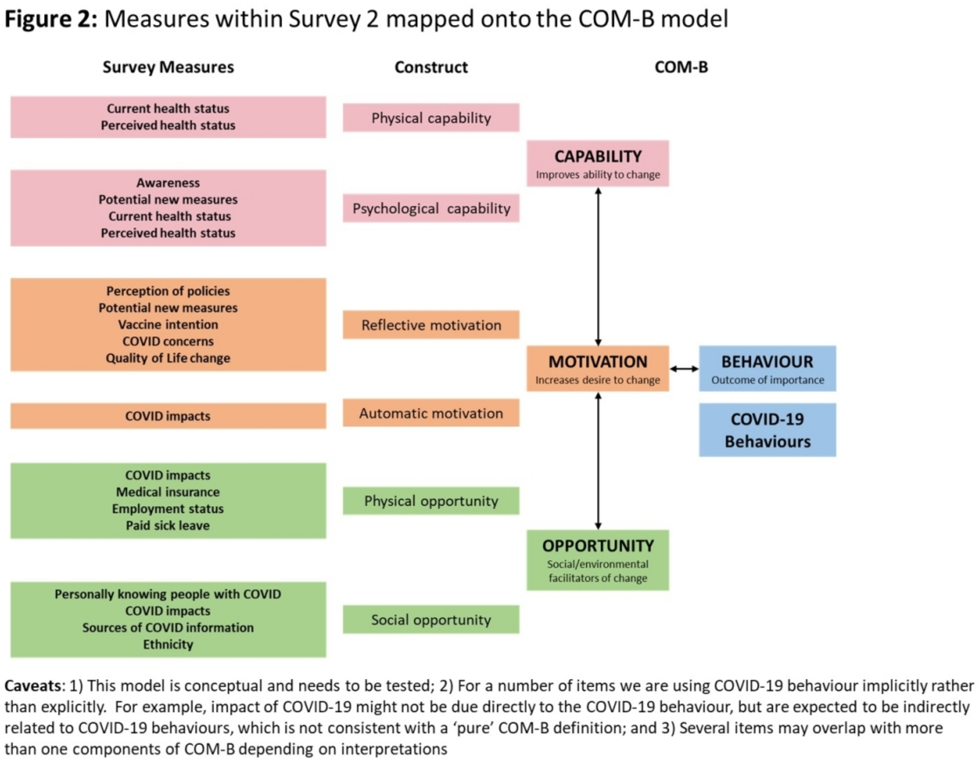

Figure 2: Measures within Survey 2 mapped onto the COM-B model

376x292mm (96 x 96 DPI)

Figure 3: Survey release timeline

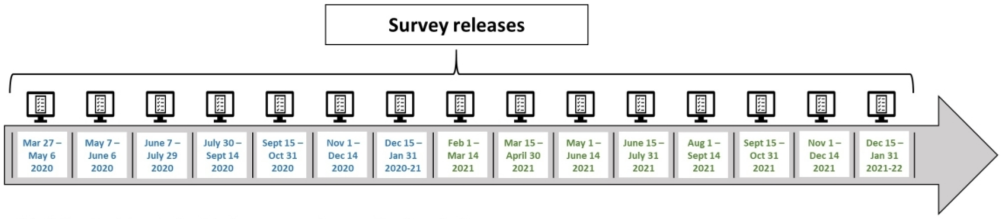

Dates in blue already have funding. Dates in green are pending current funding applications

Figure 3: Survey release timeline

522x176mm (96 x 96 DPI)

Supplementary Material

iCARE Study team

Lead investigators: Kim L. Lavoie, PhD, University of Quebec at Montreal (UQAM) and CIUSSS-NIM, CANADA; Simon L. Bacon, PhD, Concordia University and CIUSSS-NIM, CANADA.

Collaborators (in alphabetical order: Country then investigator): ABU DHABI: Zahir Vally, PhD, United Arab Emirates University; AUSTRALIA: Jacqueline Boyle, PhD, Monash University; Joanne Enticott, PhD, Monash University; Shajedur Rahman Shawon, PhD, Centre for Big Data Research in Health, UNSW Medicine; Helena Teede, MD, Monash University; AUSTRIA: Alexandra Kautzky-Willer, MD, Medizinische Universität Wien; BANGLADESH: Arobindu Dash, MS, International University of Business, Agriculture & Technology; BRAZIL: Marilia Estevam Cornelio, PhD, University of Campinas; Marlus Karsten, Universidade do Estado de Santa Catarina - UDESC; Darlan Lauricio Matte, PhD, Universidade do Estado de Santa Catarina - UDESC; CANADA: Ahmed Abou-Setta, PhD, University of Manitoba; Shawn Aaron, PhD, Ottawa Hospital Research Institute; Angela Alberga, PhD, Concordia University; Tracie Barnett, PhD, McGill University; Silvana Barone, MD, Université de Montréal; Ariane Bélanger-Gravel, PhD, Université Laval; Sarah Bernard, PhD, Université Laval; Lisa Maureen Birch, PhD, Université Laval; Susan Bondy, PhD, University of Toronto - Dalla Lana School of Public Health; Linda Booij, PhD, Concordia University; Roxane Borgès Da Silva, PhD, Université de Montréal; Jean Bourbeau, MD, McGill University; Rachel Burns, PhD, Carleton University; Tavis Campbell, PhD, University of Calgary; Linda Carlson, PhD, University of Calgary; Kim Corace, PhD, University of Ottawa; Olivier Drouin, MD, CHU Sainte-Justine/Université de Montréal; Francine Ducharme, MD, Université de Montréal; Mohsen Farhadloo, Concordia University; Carl Falk, PhD, McGill University; Richard Fleet MD, PhD, Université Laval; Michel Fournier, MSc, Direction de la Santé Publique de Montréal; Gary Garber, MD, University of Ottawa/Public Health Ontario; Lise Gauvin, PhD, Université de Montréal; Jennifer Gordon, PhD, University of Regina; Roland Grad, MD, McGill University; Samir Gupta, MD, University of Toronto; Kim Hellemans, PhD, Carleton University; Catherine Herba PhD, UQAM; Heungsun Hwang, PhD, McGill University; Keven Joyal-Desmarais, PhD, Concordia University; Lisa Kakinami, PhD, Concordia University; Eric Kennedy, PhD, York University; Sunmee Kim, PhD, University of Manitoba; Joanne Liu, PhD, McGill University; Sandra Pelaez, PhD, Université de

For peer review only - http://bmjopen.bmj.com/site/about/guidelines.xhtml

Montréal; Louise Pilote, MD, McGill University; Paul Poirier, MD, Université Laval; Justin Presseau, PhD, University of Ottawa; Eli Puterman, PhD, University of British Columbia; Joshua Rash, PhD, Memorial University; Paula AB Ribeiro, PhD, MBMC; Mohsen Sadatsafavi, PhD, University of British Columbia; Paramita Saha Chaudhuri, PhD, McGill University; Jovana Stojanovic, PhD, Concordia University; Eva Suarthana, MD, PhD, Université de Montréal / McGill University; Michael Vallis, PhD, Dalhousie University; CHILE: Nicolás Bronfman Caceres, PhD, Universidad Andrés Bello; Manuel Ortiz, PhD, Universidad de La Frontera; Paula Beatriz Repetto, PhD, Universidad Católica de Chile; COLOMBIA: Mariantonia Lemos-Hoyos, PhD, Universidad EAFIT; CYPRUS: Angelos Kassianos, PhD, University of Cyprus; DENMARK: Naja Hulvej Rod, PhD, University of Copenhagen; FRANCE: Mathieu Beraneck, PhD, Université de Paris; CNRS; Gregory Ninot, PhD, Université de Montpellier; GERMANY: Beate Ditzen, PhD, Heidelberg University; Thomas Kubiak, PhD, Mainz University; GHANA: Sam Codjoe MPhil,MSc, University of Ghana; Lily Kpobi, PhD, University of Ghana; Amos Laar, PhD, University of Ghana; INDIA: Naorem Kiranmala Devi, PhD, University of Delhi; Sanjenbam Meitei, PhD, Manipur University; Suzanne Tanya Nethan, MDS, ICMR-National Institute of Cancer Prevention & Research; Lancelot Pinto, MD, PhD, Hinduja Hospital and Medical Research Centre; Kallur Nava Saraswathy, PhD, University of Delhi; Dheeraj Tumu, MD, World Health Organization (WHO); INDONESIA: Silviana Lestari, MD, PhD, Universitas Indonesia; Grace Wangge, MD, PhD, SEAMEO Regional Center for Food and Nutrition; IRELAND: Molly Byrne, PhD, National University of Ireland, Galway; Hannah Durand, PhD, National University of Ireland, Galway; Jennifer McSharry, PhD, National University of Ireland, Galway; Oonagh Meade, PhD, National University of Ireland, Galway; Gerry Molloy, PhD, National University of Ireland, Galway; Chris Noone, PhD, National University of Ireland, Galway; ISRAEL: Hagai Levine, MD, Hebrew University; Anat Zaidman-Zait, PhD, Tel-Aviv University; ITALY: Stefania Boccia, PhD, Università Cattolica del Sacro Cuore; Ilda Hoxhaj, MD, Università Cattolica del Sacro Cuore, Stefania Paduano, MSc, PhD, University of Modena and Reggio Emilia; Valeria Raparelli, PhD, Sapienza - University of Rome; Drieda Zaçe, MD, MSc, PhDc, Università Cattolica del Sacro Cuore; JORDAN: Ala'S Aburub, PhD, Isra University; KENYA: Daniel Akunga, PhD, Kenyatta University; Richard Ayah, PhD, University of Nairobi, School Public Health; Chris Barasa, MPH, University of Nairobi, School Public Health; Pamela Miloya Godia, PhD, University of Nairobi; Elizabeth W. Kimani-Murage, PhD, African Population and Health Research Center; Nicholas Mutuku, PhD, University of Kenya; Teresa Mwoma, PhD, Kenyatta University; Violet Naanyu, PhD, Moi University; Jackim Nyamari, PhD, Kenyatta University; Hildah Oburu, PhD, Kenyatta University; Joyce Olenja, PhD, University of Nairobi;

For peer review only - http://bmjopen.bmj.com/site/about/guidelines.xhtml

Dismas Ongore, PhD, University of Nairobi; Abdhalah Ziraba, PhD, African Population and Health Research Center; MALAWI: Chiwoza Bandawe, PhD, University of Malawi; MALAYSIA: Loh Siew Yim, PhD, Faculty of medicine, University of Malaya; NEW ZEALAND: Andrea Herbert, PhD, University of Canterbury; Daniela Liggett, PhD, University of Canterbury; NIGERIA: Ademola Ajuwon, PhD, University of Ibadan; PAKISTAN: Nisar Ahmed Shar, PhD, CoPI-National Center in Big Data & Cloud Computing; Bilal Ahmed Usmani, PhD, NED University of Engineering and Technology; PERU: Rosario Mercedes Bartolini Martínez, PhD, Instituto de Investigacion Nutricional; Hilary Creed-Kanashiro, M.Phil., Instituto de Investigacion Nutricional; PORTUGAL: Paula Simão, MD, S. Pneumologia de Matosinhos; RWANDA: Pierre Claver Rutayisire, PhD, University Rwanda; SAUDI ARABIA: Abu Zeeshan Bari, PhD, Taibah University; SLOVAKIA: Iveta Nagyova, PhD, PJ Safarik University - UPJS; SOUTH AFRICA: Jason Bantjes, PhD, University of Stellenbosch; Brendon Barnes, PhD, University of Johannesburg; Bronwyne Coetzee, PhD, University of Stellenbosch; Ashraf Khagee, PhD, University of Stellenbosch; Tebogo Mothiba, PhD, University of Limpopo; Rizwana Roomaney, PhD, University of Stellenbosch; Leslie Swartz, PhD University of Stellenbosch; SOUTH KOREA: Juhee Cho, PhD, Sungkyunkwan University; Man-gyeong Lee, PhDc, Sungkyunkwan University; SWEDEN: Anne Berman, PhD, Karolinska Institutet; Nouha Saleh Stattin, MD, Karolinska Institutet; SWITZERLAND: Susanne Fischer, PhD, University of Zurich; TAIWAN: Debbie Hu, MD, MSc, Tainan Municipal Hospital; TURKEY: Yasin Kara, MD, Kanuni Sultan Süleyman Training and Research Hospital, Istanbul; Ceprail Şimşek, MD Health Science University; Bilge Üzmezoğlu, MD, University of Health Science; UGANDA: John Bosco Isunju, PhD, Makerere University School of Public Health; James Mugisha, PhD, University of Uganda; UK: Lucie Byrne-Davis, PhD, University of Manchester; Paula Griffiths, PhD, Loughborough University; Joanne Hart, PhD, University of Manchester; Will Johnson, PhD, Loughborough University; Susan Michie, PhD, University College London; Nicola Paine, PhD, Loughborough University; Emily Petherick, PhD, Loughborough University; Lauren Sherar, PhD, Loughborough University; USA: Robert M. Bilder, PhD, ABPP-CN, University of California, Los Angeles; Matthew Burg, PhD, Yale; Susan Czajkowski, PhD, NIH - National Cancer Institute; Ken Freedland, PhD, Washington University; Sherri Sheinfeld Gorin, PhD, University of Michigan; Alison Holman, PhD, University of California, Irvine; Jiyoung Lee, PhD, University of Alabama; Gilberto Lopez ScD, MA, MPH, Arizona State University and University of Rochester Medical Center; Sylvie Naar, PhD, Florida State University; Michele Okun, PhD, University of Colorado, Colorado Springs; Lynda Powell, PhD, Rush University; Sarah Pressman, PhD, University of California, Irvine; Tracey Revenson, PhD, University of New York City; John Ruiz, PhD, University of

For peer review only - http://bmjopen.bmj.com/site/about/guidelines.xhtml

Arizona; Sudha Sivaram, PhD, NIH, Center for Global Health; Johannes Thrul, PhD, Johns Hopkins; Claudia Trudel-Fitzgerald, PhD, Harvard T.H. Chan School of Public Health; Abehaw Yohannes, PhD, Azusa Pacific University.

Students (in alphabetical order): AUSTRALIA: Rhea Navani, BSc, Monash University; Kushnan Ranakombu, PhD, Monash University; BRAZIL: Daisuke Hayashi Neto, Unicamp; CANADA: Tair Ben-Porat, PhD, Tel Aviv University; Anda Dragomir, University of Quebec at Montreal (UQAM) and CIUSSS-NIM; Amandine Gagnon-Hébert, BA, UQAM; Claudia Gemme, MSc, UQAM; Vincent Gosselin Boucher, University of Quebec at Montreal (UQAM) and CIUSSS-NIM; Mahrukh Jamil, Concordia University and CIUSSS-NIM; Lisa Maria Käfer, McGill University; Ariany Marques Vieira, MSc, Concordia University; Tasfia Tasbih, Concordia University and CIUSSS-NIM; Maegan Trottier, University of Lethbridge; Robbie Woods, MSc, Concordia University; Reyhaneh Yousefi, Concordia University and CIUSSS-NIM; FRANCE: Tamila Roslyakova, Université de Montpellier; GERMANY: Lilli Priesterroth, Mainz University; ISRAEL: Shirly Edelstein, Hebrew University-Hadassah School of Public Health; Tanya Goldfrad, Hebrew University-Hadassah School of Public Health; Ruth Snir, Hebrew University-Hadassah School of Public Health; Yifat Uri, Hebrew University-Hadassah School of Public Health; NEW ZEALAND: Mohsen Alyami, University of Auckland; NIGERIA: Comfort Sanuade; SERBIA: Katarina Vojvodic, University of Belgrade.

Community Participants: CANADA: Olivia Crescenzi; Kyle Warkentin; DENMARK: Katya Grinko; INDIA: Lalita Angne; Jigisha Jain; Nikita Mathur, Syncorp Clinical Research; Anagha Mithe; Sarah Nethan, Community Empowerment Lab.

For peer review only - http://bmjopen.bmj.com/site/about/guidelines.xhtml

STANDARD PROTOCOL ITEMS: RECOMMENDATIONS FOR INTERVENTIONAL TRIALS

SPIRIT 2013 Checklist: Recommended items to address in a clinical trial protocol and related documents*

Section/item	ItemNo	Page number	Description
**Administrative information**			
Title	1	1	Descriptive title identifying the study design, population, interventions, and, if applicable, trial acronym
Trial registration	2a	n/a	Trial identifier and registry name. If not yet registered, name of intended registry
	2b	n/a	All items from the World Health Organization Trial Registration Data Set
Protocol version	3	All pages	Date and version identifier
Funding	4	1-2	Sources and types of financial, material, and other support
Roles and responsibilities	5a	1	Names, affiliations, and roles of protocol contributors
	5b	n/a	Name and contact information for the trial sponsor
	5c	2	Role of study sponsor and funders, if any, in study design; collection, management, analysis, and interpretation of data; writing of the report; and the decision to submit the report for publication, including whether they will have ultimate authority over any of these activities
	5d	9	Composition, roles, and responsibilities of the coordinating centre, steering committee, endpoint adjudication committee, data management team, and other individuals or groups overseeing the trial, if applicable (see Item 21a for data monitoring committee)

For peer review only

**Introduction**			
Background and rationale	6a	7-8	Description of research question and justification for undertaking the trial, including summary of relevant studies (published and unpublished) examining benefits and harms for each intervention
	6b	n/a	Explanation for choice of comparators
Objectives	7	8	Specific objectives or hypotheses
Trial design	8	9	Description of trial design including type of trial (eg, parallel group, crossover, factorial, single group), allocation ratio, and framework (eg, superiority, equivalence, noninferiority, exploratory)
**Methods: Participants, interventions, and outcomes**			
Study setting	9	10-11	Description of study settings (eg, community clinic, academic hospital) and list of countries where data will be collected. Reference to where list of study sites can be obtained
Eligibility criteria	10	10-11	Inclusion and exclusion criteria for participants. If applicable, eligibility criteria for study centres and individuals who will perform the interventions (eg, surgeons, psychotherapists)
Interventions	11a	n/a	Interventions for each group with sufficient detail to allow replication, including how and when they will be administered
	11b	n/a	Criteria for discontinuing or modifying allocated interventions for a given trial participant (eg, drug dose change in response to harms, participant request, or improving/worsening disease)

For peer review only

	11c	n/a	Strategies to improve adherence to intervention protocols, and any procedures for monitoring adherence (eg, drug tablet return, laboratory tests)
	11d	n/a	Relevant concomitant care and interventions that are permitted or prohibited during the trial
Outcomes	12	9-10	Primary, secondary, and other outcomes, including the specific measurement variable (eg, systolic blood pressure), analysis metric (eg, change from baseline, final value, time to event), method of aggregation (eg, median, proportion), and time point for each outcome. Explanation of the clinical relevance of chosen efficacy and harm outcomes is strongly recommended
Participant timeline	13	10-11	Time schedule of enrolment, interventions (including any run-ins and washouts), assessments, and visits for participants. A schematic diagram is highly recommended (see Figure)
Sample size	14	n/a	Estimated number of participants needed to achieve study objectives and how it was determined, including clinical and statistical assumptions supporting any sample size calculations
Recruitment	15	10-11	Strategies for achieving adequate participant enrolment to reach target sample size
**Methods: Assignment of interventions (for controlled trials)**			
Allocation:			
Sequence generation	16a	n/a	Method of generating the allocation sequence (eg, computer-generated random numbers), and list of any factors for stratification. To reduce predictability of a random sequence, details of any planned restriction (eg, blocking) should be provided in a separate document that is unavailable to those who enrol participants or assign interventions

Allocation concealment mechanism	16b	n/a	Mechanism of implementing the allocation sequence (eg, central telephone; sequentially numbered, opaque, sealed envelopes), describing any steps to conceal the sequence until interventions are assigned
Implementation	16c	n/a	Who will generate the allocation sequence, who will enrol participants, and who will assign participants to interventions
Blinding (masking)	17a	n/a	Who will be blinded after assignment to interventions (eg, trial participants, care providers, outcome assessors, data analysts), and how
	17b	n/a	If blinded, circumstances under which unblinding is permissible, and procedure for revealing a participant's allocated intervention during the trial
**Methods: Data collection, management, and analysis**			
Data collection methods	18a	10-12	Plans for assessment and collection of outcome, baseline, and other trial data, including any related processes to promote data quality (eg, duplicate measurements, training of assessors) and a description of study instruments (eg, questionnaires, laboratory tests) along with their reliability and validity, if known. Reference to where data collection forms can be found, if not in the protocol
	18b	n/a	Plans to promote participant retention and complete follow-up, including list of any outcome data to be collected for participants who discontinue or deviate from intervention protocols
Data management	19	13	Plans for data entry, coding, security, and storage, including any related processes to promote data quality (eg, double data entry; range checks for data values). Reference to where details of data management procedures can be found, if not in the protocol
Statistical methods	20a	13-14	Statistical methods for analysing primary and secondary outcomes. Reference to where other details of the statistical analysis plan can be found, if not in the protocol

	20b	13	Methods for any additional analyses (eg, subgroup and adjusted analyses)
	20c	n/a	Definition of analysis population relating to protocol non-adherence (eg, as randomised analysis), and any statistical methods to handle missing data (eg, multiple imputation)
**Methods: Monitoring**			
Data monitoring	21a	n/a	Composition of data monitoring committee (DMC); summary of its role and reporting structure; statement of whether it is independent from the sponsor and competing interests; and reference to where further details about its charter can be found, if not in the protocol. Alternatively, an explanation of why a DMC is not needed
	21b	n/a	Description of any interim analyses and stopping guidelines, including who will have access to these interim results and make the final decision to terminate the trial
Harms	22	n/a	Plans for collecting, assessing, reporting, and managing solicited and spontaneously reported adverse events and other unintended effects of trial interventions or trial conduct
Auditing	23	n/a	Frequency and procedures for auditing trial conduct, if any, and whether the process will be independent from investigators and the sponsor
**Ethics and dissemination**			
Research ethics approval	24	14	Plans for seeking research ethics committee/institutional review board (REC/IRB) approval
Protocol amendments	25	n/a	Plans for communicating important protocol modifications (eg, changes to eligibility criteria, outcomes, analyses) to relevant parties (eg, investigators, REC/IRBs, trial participants, trial registries, journals, regulators)
Consent or assent	26a	14	Who will obtain informed consent or assent from potential trial participants or authorised surrogates, and how (see Item 32)

only

For peer review only - http://bmjopen.bmj.com/site/about/guidelines.xhtml

	26b	n/a	Additional consent provisions for collection and use of participant data and biological specimens in ancillary studies, if applicable
Confidentiality	27	14	How personal information about potential and enrolled participants will be collected, shared, and maintained in order to protect confidentiality before, during, and after the trial
Declaration of interests	28	2	Financial and other competing interests for principal investigators for the overall trial and each study site
Access to data	29	16	Statement of who will have access to the final trial dataset, and disclosure of contractual agreements that limit such access for investigators
Ancillary and post-trial care	30	n/a	Provisions, if any, for ancillary and post-trial care, and for compensation to those who suffer harm from trial participation
Dissemination policy	31a	14-15	Plans for investigators and sponsor to communicate trial results to participants, healthcare professionals, the public, and other relevant groups (eg, via publication, reporting in results databases, or other data sharing arrangements), including any publication restrictions
	31b	14-15	Authorship eligibility guidelines and any intended use of professional writers
	31c	14-15	Plans, if any, for granting public access to the full protocol, participant-level dataset, and statistical code
**Appendices**			
Informed consent materials	32	n/a	Model consent form and other related documentation given to participants and authorised surrogates

For peer review only - http://bmjopen.bmj.com/site/about/guidelines.xhtml

Biological specimens	33	n/a	Plans for collection, laboratory evaluation, and storage of biological specimens for genetic or molecular analysis in the current trial and for future use in ancillary studies, if applicable

*It is strongly recommended that this checklist be read in conjunction with the SPIRIT 2013 Explanation & Elaboration for important clarification on the items. Amendments to the protocol should be tracked and dated. The SPIRIT checklist is copyrighted by the SPIRIT Group under the Creative Commons "Attribution-NonCommercial-NoDerivs 3.0 Unported" license.

For peer review only

An international assessment of the link between COVID-19-related attitudes, concerns and behaviours in relation to public health policies: Optimising policy strategies to improve health, economic and quality of life outcomes (the iCARE Study). Protocol Paper

Journal:	*BMJ Open*
Manuscript ID	bmjopen-2020-046127.R2
Article Type:	Protocol
Date Submitted by the Author:	13-Feb-2021
Complete List of Authors:	Bacon, Simon; Concordia University, Health, Kinesiology, and Applied Physiology; CIUSSS du Nord-de-l'Ile-de-Montreal, Montreal Behavioural Medicine Centre<br>Lavoie, Kim; Université du Québec à Montréal, Psychology; CIUSSS du Nord-de-l'Ile-de-Montreal, Montreal Behavioural Medicine Centre<br>Boyle, Jacqueline; Monash University, Diabetes and Vascular Medicine Unit<br>Stojanovic, Jovana; Concordia University, HKAP; CIUSSS du Nord-de-l'Ile-de-Montreal, Montreal Behavioural Medicine Centre<br>Joyal-Desmarais, Keven; Concordia University, HKAP; CIUSSS du Nord-de-l'Ile-de-Montreal, Montreal Behavioural Medicine Centre
**Primary Subject Heading**:	Public health
Secondary Subject Heading:	Communication, Epidemiology, Health policy
Keywords:	COVID-19, Health policy < HEALTH SERVICES ADMINISTRATION & MANAGEMENT, PREVENTIVE MEDICINE, PUBLIC HEALTH

SCHOLARONE™ Manuscripts

For peer review only - http://bmjopen.bmj.com/site/about/guidelines.xhtml

I, the Submitting Author has the right to grant and does grant on behalf of all authors of the Work (as defined in the below author licence), an exclusive licence and/or a non-exclusive licence for contributions from authors who are: i) UK Crown employees; ii) where BMJ has agreed a CC-BY licence shall apply, and/or iii) in accordance with the terms applicable for US Federal Government officers or employees acting as part of their official duties; on a worldwide, perpetual, irrevocable, royalty-free basis to BMJ Publishing Group Ltd ("BMJ") its licensees and where the relevant Journal is co-owned by BMJ to the co-owners of the Journal, to publish the Work in this journal and any other BMJ products and to exploit all rights, as set out in our licence.

The Submitting Author accepts and understands that any supply made under these terms is made by BMJ to the Submitting Author unless you are acting as an employee on behalf of your employer or a postgraduate student of an affiliated institution which is paying any applicable article publishing charge ("APC") for Open Access articles. Where the Submitting Author wishes to make the Work available on an Open Access basis (and intends to pay the relevant APC), the terms of reuse of such Open Access shall be governed by a Creative Commons licence – details of these licences and which Creative Commons licence will apply to this Work are set out in our licence referred to above.

Other than as permitted in any relevant BMJ Author's Self Archiving Policies, I confirm this Work has not been accepted for publication elsewhere, is not being considered for publication elsewhere and does not duplicate material already published. I confirm all authors consent to publication of this Work and authorise the granting of this licence.

For peer review only

Title: An international assessment of the link between COVID-19-related attitudes, concerns and behaviours in relation to public health policies: Optimising policy strategies to improve health, economic and quality of life outcomes (the iCARE Study). Protocol Paper.

Brief title: The iCARE study: Protocol paper

Authors: Simon L. Bacon, PhD,[a,b] Kim L. Lavoie, PhD,[a,c] Jacqueline Boyle, MD,[d,e] Jovana Stojanovic, PhD,[a,b] and Keven Joyal-Desmarais, PhD,[a,b] for the iCARE study team*

Word count: 3,415

Author affiliations:

a. Montreal Behavioural Medicine Centre (MBMC), Centre de recherche CIUSSS-NIM, 5400 Gouin West Blvd., Montreal, Quebec, H4J 1C5, Canada

b. Department of Health, Kinesiology, and Applied Physiology, Concordia University, Montreal, Canada

c. Department of Psychology, Université du Québec à Montréal (UQAM), Montreal, Canada

d. Monash Centre for Health Research and Implementation, Monash University, 43-51 Kanooka Grove, Clayton, VIC, 3168 Australia

e. School of Public Health and Preventive Medicine, Monash University, 553 St Kilda Road, Melbourne, VIC, 3004 Australia

* The complete list of iCARE Study collaborators appears in the Supplementary Material.

Study registration: N/A

Address for correspondence:

Simon L. Bacon, PhD, FTOS, FCCS, FABMR

Montreal Behavioral Medicine Centre

CIUSSS-NIM

Email: simon.bacon@concordia.ca

Twitter: @sbacon20, @mbmc_cmcm

Version 3: 12th February 2021

For peer review only

Abstract:

Introduction: In the context of a highly contagious virus with only recently approved vaccines and no cure, the key to slowing the spread of the COVID-19 disease and successfully transitioning through the phases of the pandemic, including vaccine uptake, is public adherence to rapidly evolving behaviour-based public health policies. The overall objective of the iCARE Study is to assess public awareness, attitudes, concerns, and behavioural responses to COVID-19 public health policies, and their impacts, on people around the world, and to link behavioural survey data with policy, mobility, and case data to provide behavioural science, data-driven recommendations to governments on how to optimise current policy strategies to reduce the impact of the COVID-19 pandemic.

Methods and analyses: The iCARE study (www.mbmc-cmcm.ca/covid19) utilises a multiple cross-sectional survey design to capture self-reported information on a variety of COVID-19 related variables from individuals around the globe. Survey data is captured using two data capture methods, convenience and representative sampling. This data is then linked to open access data for policies, cases, and population movement.

Ethics and Dissemination: The primary ethical approval was obtained from the co-ordinating site, the CIUSSS-NIM (REB#: 2020-2099 / 03-25-2020). This study will provide high-quality, accelerated and real-time evidence to help us understand the effectiveness of evolving country-level policies and communication strategies to reduce the spread of the COVID-19. Due to the urgency of the pandemic, results will be disseminated in a variety of ways, including policy briefs, social media posts, press releases, and through regular scientific methods.

Registration: N/A

Keywords: Evidence-based policies; Behaviour change; COVID-19

For peer review only - http://bmjopen.bmj.com/site/about/guidelines.xhtml

Strengths and limitations of this study

- This is a large, international study that has data captured from over 150 countries.
- The survey was constructed around well recognised behavioural theories and frameworks.
- The study is primarily being conducted online which may limit some of the generalisability of the data that is available, especially in lower and middle income countries.
- The primary data capture method is through snowball sampling, which is likely to create some bias in the sample. However, some of this can be adjusted using weightings from the representative samples that are being collected.
- A key strength of the study is that it has been developed to provide constructive policy and communication strategy data which can be implemented by governments to improve adherence to COVID-19 mitigation methods.

For peer review only

For peer review only - http://bmjopen.bmj.com/site/about/guidelines.xhtml

Abbreviations:

CIUSSS-NIM – Centre intégré universitaire de santé et de services sociaux du Nord-de-l’Île-de-Montréal

COM-B – Capability, Opportunity, Motivation-Behaviour Model

iCARE – International assessment of the link between COVID-19-related attitudes, concerns and behaviours in relation to public health policies

IGLS – Iterative generalized least squares

LMIC – Low- and Middle-income countries

MBMC – Montreal Behavioural Medicine Centre

MCMC – Markov chain Monte Carlo

OxCGRT – Oxford COVID-19 Government Response Tracker

UQAM – Université du Québec à Montréal

For peer review only

For peer review only - http://bmjopen.bmj.com/site/about/guidelines.xhtml

Introduction:

With only recently approved vaccines and no cure, the key to slowing the spread of COVID-19 and successfully transitioning through the phases of the pandemic, is ***public adherence*** to unprecedented and rapidly evolving **behaviour-based** public health policies (1, 2). To date, adherence to these policies has been critical to reducing the spread of COVID-19 and have ranged from personal hygiene measures (e.g., hand washing) to strict lockdown measures (e.g., business and school closures) (3-5). However, adherence to most of these policies requires making behavioural changes that may come with significant personal, social and economic costs, which may undermine their impact (6). For example, despite public health messages promoting the 'advantages' of adhering to COVID-19 mitigation measures, adherence to policies that may come with high personal costs (i.e., physical distancing) have been much poorer (54%) than for other 'less costly' behaviours like hand washing (90%) (7). Further, as we look towards changing lockdown measures, people's willingness to adhere to evolving government recommendations (e.g., school and store reopening's, receiving vaccines) will also be critical for re-engaging the economy whilst minimising the potential for future waves of the pandemic. Unfortunately, policy variations between and within countries, have created public confusion and uncertainty about government policy motives (8). In addition, governments have predominantly designed policies based on how they believe people 'should' behave and have ascribed little consideration to what we know about how people actually behave (9, 10).

Decades of behavioural science research has revealed that human behaviour is predictable and modifiable (11). Multiple factors are likely to predict why people adhere (or not) to various public health measures, which, in the context of COVID-19, can be defined using two related behaviour prediction models: 1) *The Capability, Opportunity, Motivation-Behaviour (COM-B) Model* (2, 12), which predicts that behaviour change depends on: awareness of prevention measures and the ability to enact them (capability), the belief that measures are personally relevant and important (motivation), and having the social and environmental resources required to adopt the behaviour (opportunity) (see **Figure 1a**); and 2) *The Health Beliefs Model* (13, 14), which posits that in adopting disease prevention measures, a person's belief in the personal threat(s) posed by the disease, together with a person's belief in the importance and effectiveness of recommended behaviours, will predict the likelihood a person adopting (or not) a particular behaviour (**Figure 1b**). In the context of this unprecedented health, social, and economic crisis, where the global need for adherence to

For peer review only - http://bmjopen.bmj.com/site/about/guidelines.xhtml

rapidly evolving public health policies has never been greater, our understanding of the determinants of adherence at each phase of the pandemic, and as a function of various policies, is critical for effective policy planning, communication, and effectiveness.

Insert Figure 1 about here

The overall goal of the iCARE Study is to assess public awareness, attitudes, concerns, and behavioural responses to COVID-19 public health policies, and their impacts, on people around the world (www.mbmc-cmcm.ca/covid19), and to link behavioural survey data with policy, mobility, and case data to provide behavioural science, data-driven recommendations to governments on how to optimise current policy strategies to reduce the impact of the COVID-19 pandemic worldwide. Specifically, we will address the following:

1) What are the **key individual characteristics** (e.g., sociodemographic; psychological; behavioural; physical/mental health; and economic) that **are associated with adherence** to major COVID-19 public health policies *in general* and by *country*?
2) To what extent are COVID-19 **attitudes, beliefs and concerns associated with adherence**, and how does this vary across key subgroups (e.g., age, sex, income, family/household structure, ethnic groups, those with health conditions, etc.)?
3) What are the short- and medium-term **impacts of COVID-19 and its public health policies**, and how do they vary as a function of key individual characteristics in *general* and by *country*?
4) Which policies and strategies are associated with better (and worse) adherence, are most (and least) effective at reducing infection rates, and positively impact economic growth (where appropriate)? As well as, identifying in whom these polices and strategies worked (and did not work).
5) The development of **behavioural science, data-driven, tailored recommendations,** that governments could use to optimise policy and communication strategies to improve adherence, as well as, health, economic, and quality of life outcomes.

For peer review only - http://bmjopen.bmj.com/site/about/guidelines.xhtml

Methods and analysis

Study design:

The iCARE Study is a Canadian-led, ongoing, multi-wave international study involving the collaboration of more than **190 international researchers** from over **40 countries** (see Supplementary Material). It utilises a multiple cross-sectional survey design (each approximately 6 weeks apart) to capture self-reported information on a variety of COVID-19 related variables from individuals around the globe. Survey data is captured using two data capture methods, convenience and representative sampling (see details below). This data is then coupled to open access data for policies, cases, and population movement. The study is managed by the Montreal Behavioural Medicine Centre (MBMC: a joint Centre intégré universitaire de santé et de services sociaux du Nord-de-l'Île-de-Montréal (CIUSSS-NIM) / Université du Québec à Montréal (UQAM) / Concordia University academic research and training centre).

Patient and Public Involvement (PPI) Statement

Given the significance and broad impact of the COVID-19 pandemic PPI is crucial for effective research in this area. More importantly, given the global nature of the iCARE study it has been critical to have individuals from multiple settings included in the development of the various elements and items in the survey. To this end, we consulted with over 190 multidisciplinary collaborators (including experts from the behavioural sciences, medicine and infectious disease, public health, epidemiology, statistics, and implementation science) from more than 40 countries including researchers, clinicians, students, and members of the general public in the development and design of the iCARE study (see Supplementary Material for the iCARE team). In addition, throughout our data analysis process we have engaged critical end users, including government officials, the public, the news media, in defining areas that need critical input for which the iCARE study is able to address.

The iCARE survey:

The core elements of the survey assess the following domains:

- awareness of local COVID-19 public health policies
- attitudes/beliefs about local COVID-19 policies
- behavioural responses to local COVID-19 policies

For peer review only - http://bmjopen.bmj.com/site/about/guidelines.xhtml

- perceived concerns about COVID-19
- the impacts of COVID-19 and its policies (social, occupational, economic, quality of life, physical and mental health)
- COVID-19 information sources
- COVID-19 testing and infection status
- impacts on schools and education
- physical and mental health status
- general health behaviours, including vaccine history, attitudes, and behaviours
- socio-demographics and socio-economic barriers and facilitators of adherence

Most questions are aligned with the constructs in both the COM-B (see **Figure 2**) (12) and Health Belief Models (13, 14). Questions assessing COVID-19 impacts were also chosen to facilitate data harmonisation with international COVID-19 studies involving the NIH and WHO (15). The survey is currently available in **36 languages**, making it legible to the majority of the world's population.

Insert Figure 2 about here

Though the core content of the survey is consistent throughout each release cycle, small modifications have been made as a function of the evolving nature of COVID-19 and public health policies. All surveys are open access and can be found at: https://osf.io/nswcm. Regardless of the survey content, each questionnaire is designed to take no more than 15-20 minutes to complete.

Global convenience sample: Survey participants are being recruited using online snowball sampling by all global collaborators. The online survey (LimeSurvey©) is distributed through various channels to reach as many people around the world as possible. These channels include professional networks, associations and societies; community organisations; schools and universities; hospitals and health networks; via social media; and personal contacts. The central study coordination group creates a variety of email, social media, and public facing materials for each survey round which are then translated and provided to each collaborator. There are also a series of instructional tools which

For peer review only - http://bmjopen.bmj.com/site/about/guidelines.xhtml
For peer review only

collaborators can use that provide information and examples of ways in which they can distribute the survey through their local country networks.

To date, there have been seven survey releases (April, May, June, July, September, November, and December). There are several current funding applications that are being reviewed, which if funded, would extend the data collection to eight more releases through to January 2022 (see **Figure 3**).

Insert Figure 3 about here

Representative samples in target countries: To supplement convenience sampling, we have been conducting parallel national representative sampling in countries where funds are available. Participants in each representative sample are balanced according to age, sex, province/region, education level, and income to ensure representation across these relevant variables. Representative sampling uses polling services to distribute the iCARE survey, generally with internet based sampling methods, though for certain countries, especially low- and middle-income countries (LMICs), there may be a need to conduct telephone and in-person interviews. For example in Canada, we have used Leger © polling services, who recruit participants aged 18 and over through their Léo online panel (LégerWeb.com). This panel includes over 400,000 Canadians, most of whom (60%) have been recruited within the past 10 years. Two thirds of the panel were recruited randomly by telephone, with the remainder recruited via publicity and social media. Using data from Statistics Canada, results are weighted within each province according to the sex and age of the respondents in order to make their profiles representative of the actual population within each Canadian province. Then, the weight of each province is adjusted to make it representative of their actual weight within the Canadian federation. Representative sampling in targeted countries will enable global coverage of most geographical locations and socioeconomic gradients. In addition, representative sampling will also allow us to estimate potential biases in the convenience sample data for those countries.

Additional data sources

The Oxford COVID-19 Government Response Tracker (16, 17) systematically collects publicly available information on a variety of indicators of COVID-19 related government policy responses. These policies are then accumulated to provide a variety of indexes as estimates of the total response of an

For peer review only - http://bmjopen.bmj.com/site/about/guidelines.xhtml

individual country. *Google Mobility Data* (18) provides user mobility trends over time by country and region across different categories of places (e.g., retail, groceries, parks, transit stations, workplaces, and residential), and generates regular "Community Mobility Reports" presented by location. They report the percent change in visits to places like grocery stores and parks within a geographic area. These datasets show how visits and length of stay at different places change compared to baseline. Datasets show trends over several months with the most recent data reflecting the last 2-3 days. *Johns Hopkins Coronavirus Resource Center* (19, 20) has been tracking country-level (and province/state for Canada and the US) case, death and recovery data since the start of the pandemic, and the website is updated multiple times a day. In addition, they provide testing data for US states. The data is drawn from multiple sites.

Progress to date:

Convenience sampling

Survey 1 of the global convenience sample began on March 27, 2020. When it closed on May 6 we had received surveys from **28,651** people in **137 countries**, including more than 1,000 responses from 4 countries and more than 500 responses from 10 additional countries. Survey 2 of the global convenience sample was launched on May 5, 2020. When it closed on June 8 we had received surveys from **12,576** people in **124** countries, including more than 500 responses from 7 countries. Survey 3 of the global convenience sample was launched on June 8, 2020. When it closed on July 22 we had received surveys from **7,652** people in **100** countries, including more than 500 responses from 3 countries. Survey 4 of the global convenience sample was launched on July 22, 2020. When it closed on September 15, 2020 we had received surveys from **4,102** people in **81** countries, including more than 500 responses from 2 countries. Survey 5 of the global convenience sample was launched on September 15, 2020. When it closed on November 3, 2020 we had received surveys from **3,404** people in **87** countries, including more than 500 responses from 2 countries. Survey 6 of the global convenience sample was launched on November 3, 2020. When it closed on December 15, 2020 we had received surveys from **2,451** people in **73** countries, including more than 500 responses from 1 country.

Representative sampling

For peer review only - http://bmjopen.bmj.com/site/about/guidelines.xhtml

To date, seven rounds of representative sampling have been captured. Three of these have occurred in Canada (Survey 1: April 9-20, n=3,003, Survey 3: June 4-17, n=3,005, and Survey 6: October 28-November 10, n=3,005), two in Australia (Survey 2: May 1-5, n=1,005 and Survey 3: July 1-7, n=1,051) and one each in the UK (Survey 1: April 3-30, n=2,056) and Ireland (Survey 3: June 22-July 15, 2020, n=1,000). Current funding will allow us to capture another 2 samples in Canada along with samples from the US, Italy, and Colombia. Additional samples will be captured dependent on funding.

Data harmonization: Initially, all data sources will be aggregated at the country level, as a function of available data. However, for those with limited data it might be at the level of continent and for those with large amounts of data we may also be able to provide data at the level of region. Data sources will be tagged based on the date when each participant completed the survey. A series of generalised linear models will be developed to estimate systematic differences in responses between sexes, ethnicities, age-groups, essential worker status, and other key sociodemographic variables. Patterns of missing data will be examined and, where appropriate, accounted for by using multiple imputation techniques (21, 22). In countries where there is sufficient data in the convenience sample, we will apply weights to allow the data to provide national approximations (23-25).

Statistical analyses: With a study of this magnitude, it is impossible to detail all possible analyses that could be conducted, as these will vary based on the specific questions that might be received from governments or researcher partners. However, the following section provides a high-level overview of the kinds of 'basic' analytical strategies that will be conducted with the data. Descriptive analyses, including general linear models or logistic regressions, of the survey data will be provided to explore trends in the main areas represented in the survey. Where possible, the psychometric properties of the various elements of the survey will be explored. This will also include a variety of clustering techniques, e.g., principal components analyses (PCA) or factor analyses, to create appropriate sub-scales. For instance, to cluster and reduce the dimensionality of the COVID-19 impact questions for Surveys 2 to 4, we performed a PCA on the polychoric correlation matrix of the COVID-19 impacts variables. We used an orthogonal (varimax) rotation in order to distribute the component loadings. We identified different impact components based on the Kaiser criterion (eigenvalue >1.0) (26), scree plot, component loadings (> 0.4) and components interpretability. For the main study questions (see above), with the magnitude and complexity of the data that is being captured a number of different

For peer review only - http://bmjopen.bmj.com/site/about/guidelines.xhtml

multilevel modelling techniques will be used. As an example, exploratory iterative generalized least squares (IGLS (27)) models followed by Markov chain Monte Carlo (MCMC) estimation for some models will likely be used (28). Briefly, this is a Bayesian simulation approach which (after assigning starting values and prior distributions) sequentially samples subsets of parameters from their conditional posterior distributions given current values of the other parameters. This is a very flexible approach used by other groups with comparable data (e.g., NCD-RisC (29)). For instance, using this approach we are evaluating how the perception of government recommendations and the population's behaviour regarding facemasks wearing varies according to the date of policy implementation in five targeted countries (Canada, USA, Colombia, Brazil and France) and how this then tracks onto case rates.

For the **representative samples**, appropriate link functions will be tested and used, with the polling company's sampling weights being employed (23-25). All the national representative data will leverage the global data, by pooling all the available information (at any given point in time) and extending our models into a multilevel framework with random effects (intercepts and slopes) at the country levels. By essentially borrowing information from the other countries, this approach will improve the power to obtain robust and precise estimates for any singular country (23, 30). In addition, where possible, we will leverage the representative samples to be able to validate the 'representativeness' of the data captured in the global sample. These analyses may provide insights into potential areas of bias and so that potential further weightings could be applied to the global sample.

Ethics and dissemination

Ethics approval

The REB at the co-ordinating study site CIUSSS-NIM provides the primary ethical approval (REB#: 2020-2099 / 03-25-2020). Online consent is provided by participants prior to completing the survey. No personal identifying information is collected from any participant. In addition, several of the collaborating sites have also obtained ethical approval to distribute the survey within their country or institution, though this is not required.

Knowledge translation (KT)

For peer review only - http://bmjopen.bmj.com/site/about/guidelines.xhtml

For peer review only

Due to the evolving nature of the COVID-19 pandemic, outputs from analyses will be disseminated in a variety of ways. Regular updates will be posted to the iCARE website (www.icarestudy.com) and disseminated through the Montreal Behavioural Medicine Centre social media outlets (https://www.facebook.com/CMCMMBMC; https://twitter.com/mbmc_cmcm; https://www.instagram.com/mbmc_cmcm/). Where appropriate press releases and news media will be targeted. Of note our study has already received a great deal of media attention, with more than 75 print, radio and television interviews across the globe (as of October 20, 2020; see https://mbmc-cmcm.ca/covid19/media/ for full coverage). Within Canada, we are partnering with the Royal Society of Canada's COVID-19 Task Force to reach the general public, government and national media. Finally, we will also release results through traditional scientific methods, e.g., journal articles and conference presentations. For example, Survey 1 data was presented at the International Behavioural Trials Network Global 2020 Virtual meeting (see https://www.ibtnetwork.org/conference/virtual2020/video-session-2/).

Interpretation

This study will provide high-quality, accelerated and real-time evidence to help us understand the differing impacts of COVID-19 policies, strategies, and communication around the world. It will provide evidence for the effectiveness of evolving policies implemented to reduce the spread of the virus – both in general and among key sub-groups (e.g., younger vs older, ethnic minorities, those with health conditions). The study will also generate evolving evidence to support public health planning, decision-making and responses around the world, including low and middle-income countries. Examples of the results to date can be found at https://mbmc-cmcm.ca/covid19/research/stats/ and https://mbmc-cmcm.ca/covid19/research/infog/. Of note, the iCARE study has provided data to the Canadian (Federal), Irish, Province of Ontario (Canada), and State of Victoria (Australia) governments, covering polices ranging from facemasks, contact tracing applications, and COVID-19 vaccine uptake.

Limitations: The main limitation of the study is that the survey is being conducted online. Though there is generally good internet access for most high income countries and even some LMIC's (e.g., India), some LMICs have limited access in certain areas and within certain population sub-groups. This coupled with the convenience sampling method, means that there may be some degree of sample

For peer review only - http://bmjopen.bmj.com/site/about/guidelines.xhtml

bias. Though some of this can be adjusted for based on the representative sampling data, it can't be eliminated completely. Moreover, the fact that the iCARE survey is available in 36 languages means that certain marginalized groups (e.g., immigrants to certain countries, like Canada, the US and France, which are highly represented) will likely be able to complete the survey in their native language. This may help increase participation among those who might otherwise be excluded due to language barriers. Another limitation is the fact that we will be conducting correlation analyses. Though we will be using some sophisticated analytical modelling we can't derive direct causative relationships from the study. However, our main interest is in temporal changes in attitudes and behaviours as the pandemic evolves, so analysing repeated cross sectional cohorts still allows us to meet our study objectives.

Conclusion: Ultimately, this study will help us understand what public health policies and strategies are working, where, and for whom, which can inform changes (improvements) in policy strategy and communication to help mitigate the spread of COVID-19, especially as countries are now starting to cycle through various waves of the pandemic, and its physical/mental health, social, economic and quality of life impacts**.**

Data-sharing statement

All completed survey data is anonymous and variables are collected and coded in a way that it would not be possible to identify any specific individual within the survey. Study collaborators are able to obtain access to the data through a standard Research Materials Distribution Agreement (RMDA: see https://mbmc-cmcm.ca/covid19/research/ and http://www.osf.io/nswcm). Sub-analyses of the iCARE data are logged (https://mbmc-cmcm.ca/covid19/apl/) and are openly searchable (https://mbmc-cmcm.ca/covid19/apl/log/).

For peer review only - http://bmjopen.bmj.com/site/about/guidelines.xhtml

Contributors statement:

All authors contributed to the manuscript including: contributing substantially to conception and design of the study (SLB, KLL, JB, JS, and KJD); drafting the article and revising it critically for important intellectual content (SLB, KLL, JB, JS, and KJD); providing final approval of the version to be published (SLB, KLL, JB, JS, and KJD); and acting as guarantors of the work (SLB, KLL, JB, JS, and KJD).

Competing interests:

Dr. Bacon has received consultancy fees from Merck for the development of behavior change continuing education modules, speaker fees from Novartis and Janssen, and has served on advisory boards for Bayer, Sanofi, and Sojecci Inc, none of which are related to the current article.

Dr. Lavoie has served on the advisory board for Schering-Plough, Takeda, AbbVie, Almirall, Janssen, GSK, Boehringer Ingelheim (BI), and Sojecci Inc, and has received sponsorship for investigator-generated research grants from GlaxoSmithKline (GSK) and AbbVie, speaker fees from GSK, Astra-Zeneca, Astellas, Novartis, Takeda, AbbVie, Merck, Boehringer Ingelheim, Bayer, Pfizer and Air Liquide, and support for educational materials from Merck, none of which are related to the current article.

Drs. Boyle, Stojanovic, and Joyal-Desmarais have no competing interests to declare.

Funding statement:

The primary source of funding for the iCARE study has been primarily through re-directed funding associated with Montreal Behavioural Medicine Centre, including funds from a Canadian Institutes of Health Research-Strategy for Patient Oriented Research Mentoring Chair (SMC-151518, PI: Dr. Simon L. Bacon), a Fonds de Recherche du Québec: Santé Chair (251618, PI: Dr. Simon L. Bacon), a UQAM Research Chair (1471, PI: Dr. Kim L Lavoie), and Fonds de Recherche du Québec: Santé Senior Research Award (34757, PI: Dr. Kim L Lavoie). The Canadian representative sampling will be funded by the Canadian Institutes of Health Research (MS3-173099, PI: Simon L. Bacon) and the Fonds de Recherche du Québec: Société et Culture (2019-SE1-252541, PI: Dr. Simon L. Bacon). The Australian representative sampling was funded by Monash University and indirectly by the National Health and Medical Research Council and the Medical Research Future Fund (2579, PIs: Drs. Helena Teede and

For peer review only - http://bmjopen.bmj.com/site/about/guidelines.xhtml

Jacqueline Boyle). The Irish representative sampling was funded by the Health Research Board and the Irish Research Council (COV19-2020-097, PI: Dr. Gerard J. Molloy). The UK representative sampling was funded by CALIBRE research funding, provided by Loughborough University, UK (5705, PI: Dr. Nicola J. Paine). None of the funders were involved in the study design.

For peer review only

For peer review only - http://bmjopen.bmj.com/site/about/guidelines.xhtml

References

1. Anderson RM, Heesterbeek H, Klinkenberg D, Hollingsworth TD. How will country-based mitigation measures influence the course of the COVID-19 epidemic? Lancet. 2020;395:931-34.

2. West R, Michie S, Rubin G, Amlot R. Applying principles of behaviour change to reduce SARS-CoV-2 transmission. Nature Human Behaviour. 2020;https://doi.org/10.1038/s41562-020-0887-9.

3. Jarvis CI, Van Zandvoort K, Gimma A, Prem K, Klepac P, Rubin GJ, et al. Quantifying the impact of physical distance measures on the transmission of COVID-19 in the UK. BMC Med. 2020;18(1):124.

4. Davies NG, Kucharski AJ, Eggo RM, Gimma A, Edmunds WJ. Effects of non-pharmaceutical interventions on COVID-19 cases, deaths, and demand for hospital services in the UK: a modelling study. Lancet Public Health. 2020;5(7):e375-e85.

5. Ngonghala CN, Iboi E, Eikenberry S, Scotch M, MacIntyre CR, Bonds MH, et al. Mathematical assessment of the impact of non-pharmaceutical interventions on curtailing the 2019 novel Coronavirus. Math Biosci. 2020;325:108364.

6. Van Bavel J, Baicker K, PS. B, al. E. Using social and behavioural science to support COVID-19 pandemic response. Nature Human Behaviour. 2020.

7. Amárach Research. Impact of Coronavirus - Week 3. 2020;Available at https://amarach.com/news-blog-articles/impact-of-coronavirus-week-3.html: Accessed 27th April 2020.

8. West R, Michie S, Rubin GJ, Amlôt R. Applying principles of behaviour change to reduce SARS-CoV-2 transmission. Nature Human Behaviour. 2020.

9. Milijkovic D. Rational choice and irrational individuals or simply an irrational theory: A critical review of the hypothesis of perfect rationality. Journal of Socio-economics. 2005;34:621-34.

10. Afif Z, Islan W, Calvo-Gonzalez O, Dalton A. Behavioral Science Around the World: Profiles of 10 Countries (English). . eMBeD brief 2018;Washington, D.C.:World Bank Group.

11. Michie S, West R, Campbell R, Brown J, Gainforth H. ABC of Behaviour Change Theories. UK. : Silverback Publishing; 2014.

12. Michie S, van Stralen M, West R. The Behavior Change Wheel: a new method for characterizing and designing behavior change interventions. Implementation Science. 2011;6:42.

13. Rosenstock I, Strecher V, Becker M. Social learning theory and the health belief model. Health Education Quarterly. 1971;15(2):175-83. .

For peer review only - http://bmjopen.bmj.com/site/about/guidelines.xhtml

14. Rosenstock I. The health belief model and preventive health behavior. Health Education Monographs. 1974;2:354-86.

15. National Institutes of Health OoBSSRO. https://www.nlm.nih.gov/dr2/COVID-19_BSSR_Research_Tools.pdf. 2020.

16. Oxford University. http://www.ox.ac.uk/news/2020-03-25-oxford-university-launches-world-s-first-covid-19-government-response-tracker 2020 [

17. Hale T, Webster S, Petherick A, Phillips T, Kira B. Oxford COVID-19 Government Response Tracker, . Blavatnik School of Government 2020.

18. Google. https://www.google.com/covid19/mobility/ 2020 [

19. Dong E, Du H, Gardner L. An interactive web-based dashboard to track COVID-19 in real time. The Lancet Infectious Diseases. 2020;20(5):533-4.

20. Johns Hopkins. https://coronavirus.jhu.edu/map.html. 2020.

21. Harrell FE. Regression Modeling Strategies. New York: Springer; 2001.

22. Kleinke K, Reinecke J, Salfrán D, Spiess M. Applied Multiple Imputation. Advantages, Pitfalls, New Developments and Applications in R. New York, NY: Springer; 2020.

23. Valliant R, Dever JA, Kreuter F. Practical Tools for Designing and Weighting Survey Samples. New York, NY: Springer; 2018.

24. Bethlehem J. Weighting. In: Lavrakas PJ, editor. Encyclopedia of Survey Research Methods. Thousand Oaks, California: SAGE Publications, Inc.; 2008. p. 958-60.

25. Heeringa S, West B, Berglund P. Applied Survey Data Analysis. New York: Chapman and Hall/CRC; 2017.

26. Kaiser HF. The Application of Electronic Computers to Factor Analysis. Educational and Psychological Measurement. 1960;20(1):141-51.

27. Goldstein H. Restricted unbiased iterative generalized least-squares estimation. Biometrika. 1989;76(3):622-3.

28. Browne WJ. MCMC Estimation in MLwiN v3.03. University of Bristol: Centre for Multilevel Modelling; 2019.

29. Bixby H, Bentham J, Zhou B, Di Cesare M, Paciorek CJ, Bennett JE, et al. Rising rural body-mass index is the main driver of the global obesity epidemic in adults. Nature. 2019;569(7755):260-4.

30. Skinner C, Mason B. Weighting in the regression analysis of survey data with a cross-national application. Canadian Journal of Statistics. 2012;40(4):697-711.

For peer review only - http://bmjopen.bmj.com/site/about/guidelines.xhtml

For peer review only

For peer review only - http://bmjopen.bmj.com/site/about/guidelines.xhtml

Figure Legends

Figure 1: The theoretical models underpinning the behavioural responses to COVID-19

Figure 2: Measures within Survey 2 mapped onto the COM-B model

Figure 3: Survey release timeline

For peer review only

For peer review only - http://bmjopen.bmj.com/site/about/guidelines.xhtml

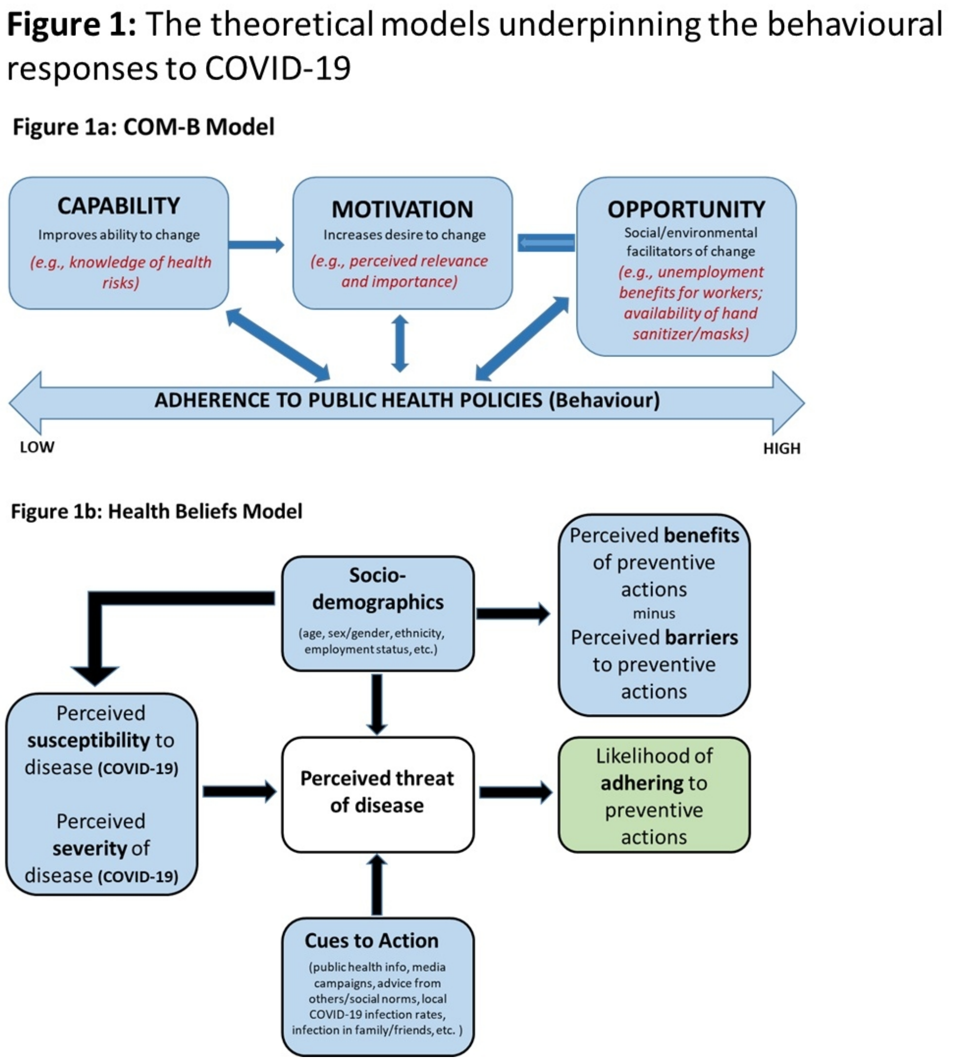

Figure 1: The theoretical models underpinning the behavioural responses to COVID-19

269x287mm (96 x 96 DPI)

Figure 2: Measures within Survey 2 mapped onto the COM-B model

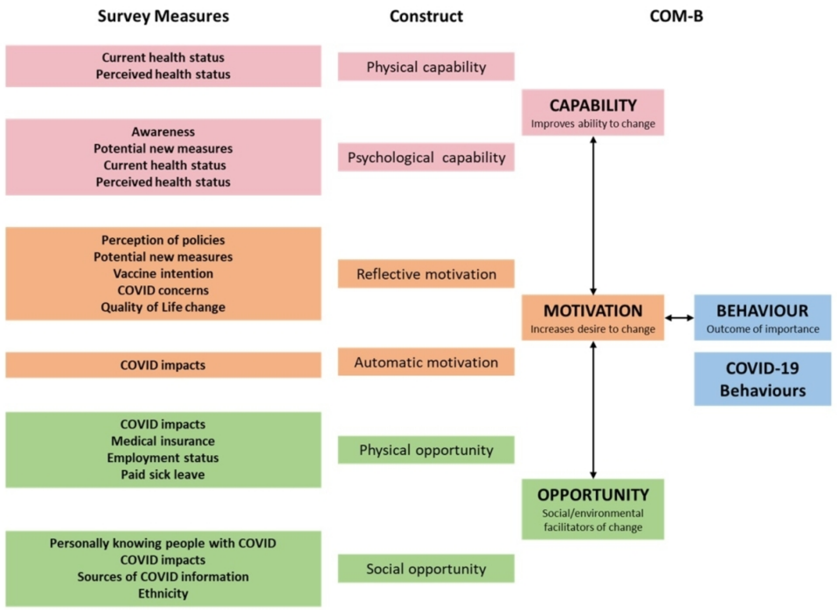

Caveats: 1) This model is conceptual and needs to be tested; 2) For a number of items we are using COVID-19 behaviour implicitly rather than explicitly. For example, impact of COVID-19 might not be due directly to the COVID-19 behaviour, but are expected to be indirectly related to COVID-19 behaviours, which is not consistent with a 'pure' COM-B definition; and 3) Several items may overlap with more than one components of COM-B depending on interpretations

Figure 2: Measures within Survey 2 mapped onto the COM-B model

376x292mm (96 x 96 DPI)

Figure 3: Survey release timeline

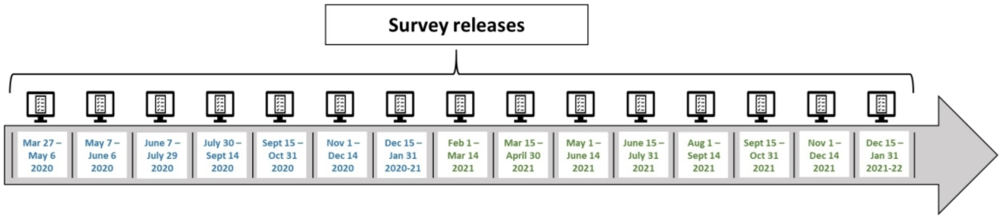

Dates in blue already have funding. Dates in green are pending current funding applications

Figure 3: Survey release timeline

522x176mm (96 x 96 DPI)

Supplementary Material

iCARE Study team

Lead investigators: Kim L. Lavoie, PhD, University of Quebec at Montreal (UQAM) and CIUSSS-NIM, CANADA; Simon L. Bacon, PhD, Concordia University and CIUSSS-NIM, CANADA.

Collaborators (in alphabetical order: Country then investigator): ABU DHABI: Zahir Vally, PhD, United Arab Emirates University; AUSTRALIA: Jacqueline Boyle, PhD, Monash University; Joanne Enticott, PhD, Monash University; Shajedur Rahman Shawon, PhD, Centre for Big Data Research in Health, UNSW Medicine; Helena Teede, MD, Monash University; AUSTRIA: Alexandra Kautzky-Willer, MD, Medizinische Universität Wien; BANGLADESH: Arobindu Dash, MS, International University of Business, Agriculture & Technology; BRAZIL: Marilia Estevam Cornelio, PhD, University of Campinas; Marlus Karsten, Universidade do Estado de Santa Catarina - UDESC; Darlan Lauricio Matte, PhD, Universidade do Estado de Santa Catarina - UDESC; CANADA: Ahmed Abou-Setta, PhD, University of Manitoba; Shawn Aaron, PhD, Ottawa Hospital Research Institute; Angela Alberga, PhD, Concordia University; Tracie Barnett, PhD, McGill University; Silvana Barone, MD, Université de Montréal; Ariane Bélanger-Gravel, PhD, Université Laval; Sarah Bernard, PhD, Université Laval; Lisa Maureen Birch, PhD, Université Laval; Susan Bondy, PhD, University of Toronto - Dalla Lana School of Public Health; Linda Booij, PhD, Concordia University; Roxane Borgès Da Silva, PhD, Université de Montréal; Jean Bourbeau, MD, McGill University; Rachel Burns, PhD, Carleton University; Tavis Campbell, PhD, University of Calgary; Linda Carlson, PhD, University of Calgary; Kim Corace, PhD, University of Ottawa; Olivier Drouin, MD, CHU Sainte-Justine/Université de Montréal; Francine Ducharme, MD, Université de Montréal; Mohsen Farhadloo, Concordia University; Carl Falk, PhD, McGill University; Richard Fleet MD, PhD, Université Laval; Michel Fournier, MSc, Direction de la Santé Publique de Montréal; Gary Garber, MD, University of Ottawa/Public Health Ontario; Lise Gauvin, PhD, Université de Montréal; Jennifer Gordon, PhD, University of Regina; Roland Grad, MD, McGill University; Samir Gupta, MD, University of Toronto; Kim Hellemans, PhD, Carleton University; Catherine Herba PhD, UQAM; Heungsun Hwang, PhD, McGill University; Keven Joyal-Desmarais, PhD, Concordia University; Lisa Kakinami, PhD, Concordia University; Eric Kennedy, PhD, York University; Sunmee Kim, PhD, University of Manitoba; Joanne Liu, PhD, McGill University; Sandra Pelaez, PhD, Université de

For peer review only - http://bmjopen.bmj.com/site/about/guidelines.xhtml

Montréal; Louise Pilote, MD, McGill University; Paul Poirier, MD, Université Laval; Justin Presseau, PhD, University of Ottawa; Eli Puterman, PhD, University of British Columbia; Joshua Rash, PhD, Memorial University; Paula AB Ribeiro, PhD, MBMC; Mohsen Sadatsafavi, PhD, University of British Columbia; Paramita Saha Chaudhuri, PhD, McGill University; Jovana Stojanovic, PhD, Concordia University; Eva Suarthana, MD, PhD, Université de Montréal / McGill University; Michael Vallis, PhD, Dalhousie University; CHILE: Nicolás Bronfman Caceres, PhD, Universidad Andrés Bello; Manuel Ortiz, PhD, Universidad de La Frontera; Paula Beatriz Repetto, PhD, Universidad Católica de Chile; COLOMBIA: Mariantonia Lemos-Hoyos, PhD, Universidad EAFIT; CYPRUS: Angelos Kassianos, PhD, University of Cyprus; DENMARK: Naja Hulvej Rod, PhD, University of Copenhagen; FRANCE: Mathieu Beraneck, PhD, Université de Paris; CNRS; Gregory Ninot, PhD, Université de Montpellier; GERMANY: Beate Ditzen, PhD, Heidelberg University; Thomas Kubiak, PhD, Mainz University; GHANA: Sam Codjoe MPhil,MSc, University of Ghana; Lily Kpobi, PhD, University of Ghana; Amos Laar, PhD, University of Ghana; INDIA: Naorem Kiranmala Devi, PhD, University of Delhi; Sanjenbam Meitei, PhD, Manipur University; Suzanne Tanya Nethan, MDS, ICMR-National Institute of Cancer Prevention & Research; Lancelot Pinto, MD, PhD, Hinduja Hospital and Medical Research Centre; Kallur Nava Saraswathy, PhD, University of Delhi; Dheeraj Tumu, MD, World Health Organization (WHO); INDONESIA: Silviana Lestari, MD, PhD, Universitas Indonesia; Grace Wangge, MD, PhD, SEAMEO Regional Center for Food and Nutrition; IRELAND: Molly Byrne, PhD, National University of Ireland, Galway; Hannah Durand, PhD, National University of Ireland, Galway; Jennifer McSharry, PhD, National University of Ireland, Galway; Oonagh Meade, PhD, National University of Ireland, Galway; Gerry Molloy, PhD, National University of Ireland, Galway; Chris Noone, PhD, National University of Ireland, Galway; ISRAEL: Hagai Levine, MD, Hebrew University; Anat Zaidman-Zait, PhD, Tel-Aviv University; ITALY: Stefania Boccia, PhD, Università Cattolica del Sacro Cuore; Ilda Hoxhaj, MD, Università Cattolica del Sacro Cuore, Stefania Paduano, MSc, PhD, University of Modena and Reggio Emilia; Valeria Raparelli, PhD, Sapienza - University of Rome; Drieda Zaçe, MD, MSc, PhDc, Università Cattolica del Sacro Cuore; JORDAN: Ala'S Aburub, PhD, Isra University; KENYA: Daniel Akunga, PhD, Kenyatta University; Richard Ayah, PhD, University of Nairobi, School Public Health; Chris Barasa, MPH, University of Nairobi, School Public Health; Pamela Miloya Godia, PhD, University of Nairobi; Elizabeth W. Kimani-Murage, PhD, African Population and Health Research Center; Nicholas Mutuku, PhD, University of Kenya; Teresa Mwoma, PhD, Kenyatta University; Violet Naanyu, PhD, Moi University; Jackim Nyamari, PhD, Kenyatta University; Hildah Oburu, PhD, Kenyatta University; Joyce Olenja, PhD, University of Nairobi;

For peer review only - http://bmjopen.bmj.com/site/about/guidelines.xhtml
For peer review only

Dismas Ongore, PhD, University of Nairobi; Abdhalah Ziraba, PhD, African Population and Health Research Center; MALAWI: Chiwoza Bandawe, PhD, University of Malawi; MALAYSIA: Loh Siew Yim, PhD, Faculty of medicine, University of Malaya; NEW ZEALAND: Andrea Herbert, PhD, University of Canterbury; Daniela Liggett, PhD, University of Canterbury; NIGERIA: Ademola Ajuwon, PhD, University of Ibadan; PAKISTAN: Nisar Ahmed Shar, PhD, CoPI-National Center in Big Data & Cloud Computing; Bilal Ahmed Usmani, PhD, NED University of Engineering and Technology; PERU: Rosario Mercedes Bartolini Martínez, PhD, Instituto de Investigacion Nutricional; Hilary Creed-Kanashiro, M.Phil., Instituto de Investigacion Nutricional; PORTUGAL: Paula Simão, MD, S. Pneumologia de Matosinhos; RWANDA: Pierre Claver Rutayisire, PhD, University Rwanda; SAUDI ARABIA: Abu Zeeshan Bari, PhD, Taibah University; SLOVAKIA: Iveta Nagyova, PhD, PJ Safarik University - UPJS; SOUTH AFRICA: Jason Bantjes, PhD, University of Stellenbosch; Brendon Barnes, PhD, University of Johannesburg; Bronwyne Coetzee, PhD, University of Stellenbosch; Ashraf Khagee, PhD, University of Stellenbosch; Tebogo Mothiba, PhD, University of Limpopo; Rizwana Roomaney, PhD, University of Stellenbosch; Leslie Swartz, PhD University of Stellenbosch; SOUTH KOREA: Juhee Cho, PhD, Sungkyunkwan University; Man-gyeong Lee, PhDc, Sungkyunkwan University; SWEDEN: Anne Berman, PhD, Karolinska Institutet; Nouha Saleh Stattin, MD, Karolinska Institutet; SWITZERLAND: Susanne Fischer, PhD, University of Zurich; TAIWAN: Debbie Hu, MD, MSc, Tainan Municipal Hospital; TURKEY: Yasin Kara, MD, Kanuni Sultan Süleyman Training and Research Hospital, Istanbul; Ceprail Şimşek, MD Health Science University; Bilge Üzmezoğlu, MD, University of Health Science; UGANDA: John Bosco Isunju, PhD, Makerere University School of Public Health; James Mugisha, PhD, University of Uganda; UK: Lucie Byrne-Davis, PhD, University of Manchester; Paula Griffiths, PhD, Loughborough University; Joanne Hart, PhD, University of Manchester; Will Johnson, PhD, Loughborough University; Susan Michie, PhD, University College London; Nicola Paine, PhD, Loughborough University; Emily Petherick, PhD, Loughborough University; Lauren Sherar, PhD, Loughborough University; USA: Robert M. Bilder, PhD, ABPP-CN, University of California, Los Angeles; Matthew Burg, PhD, Yale; Susan Czajkowski, PhD, NIH - National Cancer Institute; Ken Freedland, PhD, Washington University; Sherri Sheinfeld Gorin, PhD, University of Michigan; Alison Holman, PhD, University of California, Irvine; Jiyoung Lee, PhD, University of Alabama; Gilberto Lopez ScD, MA, MPH, Arizona State University and University of Rochester Medical Center; Sylvie Naar, PhD, Florida State University; Michele Okun, PhD, University of Colorado, Colorado Springs; Lynda Powell, PhD, Rush University; Sarah Pressman, PhD, University of California, Irvine; Tracey Revenson, PhD, University of New York City; John Ruiz, PhD, University of

For peer review only - http://bmjopen.bmj.com/site/about/guidelines.xhtml

Arizona; Sudha Sivaram, PhD, NIH, Center for Global Health; Johannes Thrul, PhD, Johns Hopkins; Claudia Trudel-Fitzgerald, PhD, Harvard T.H. Chan School of Public Health; Abehaw Yohannes, PhD, Azusa Pacific University.

Students (in alphabetical order): AUSTRALIA: Rhea Navani, BSc, Monash University; Kushnan Ranakombu, PhD, Monash University; BRAZIL: Daisuke Hayashi Neto, Unicamp; CANADA: Tair Ben-Porat, PhD, Tel Aviv University; Anda Dragomir, University of Quebec at Montreal (UQAM) and CIUSSS-NIM; Amandine Gagnon-Hébert, BA, UQAM; Claudia Gemme, MSc, UQAM; Vincent Gosselin Boucher, University of Quebec at Montreal (UQAM) and CIUSSS-NIM; Mahrukh Jamil, Concordia University and CIUSSS-NIM; Lisa Maria Käfer, McGill University; Ariany Marques Vieira, MSc, Concordia University; Tasfia Tasbih, Concordia University and CIUSSS-NIM; Maegan Trottier, University of Lethbridge; Robbie Woods, MSc, Concordia University; Reyhaneh Yousefi, Concordia University and CIUSSS-NIM; FRANCE: Tamila Roslyakova, Université de Montpellier; GERMANY: Lilli Priesterroth, Mainz University; ISRAEL: Shirly Edelstein, Hebrew University-Hadassah School of Public Health; Tanya Goldfrad, Hebrew University-Hadassah School of Public Health; Ruth Snir, Hebrew University-Hadassah School of Public Health; Yifat Uri, Hebrew University-Hadassah School of Public Health; NEW ZEALAND: Mohsen Alyami, University of Auckland; NIGERIA: Comfort Sanuade; SERBIA: Katarina Vojvodic, University of Belgrade.

Community Participants: CANADA: Olivia Crescenzi; Kyle Warkentin; DENMARK: Katya Grinko; INDIA: Lalita Angne; Jigisha Jain; Nikita Mathur, Syncorp Clinical Research; Anagha Mithe; Sarah Nethan, Community Empowerment Lab.

For peer review only

For peer review only - http://bmjopen.bmj.com/site/about/guidelines.xhtml

STANDARD PROTOCOL ITEMS: RECOMMENDATIONS FOR INTERVENTIONAL TRIALS

SPIRIT 2013 Checklist: Recommended items to address in a clinical trial protocol and related documents*

Section/item	ItemNo	Page number	Description
**Administrative information**			
Title	1	1	Descriptive title identifying the study design, population, interventions, and, if applicable, trial acronym
Trial registration	2a	n/a	Trial identifier and registry name. If not yet registered, name of intended registry
	2b	n/a	All items from the World Health Organization Trial Registration Data Set
Protocol version	3	All pages	Date and version identifier
Funding	4	1-2	Sources and types of financial, material, and other support
Roles and responsibilities	5a	1	Names, affiliations, and roles of protocol contributors
	5b	n/a	Name and contact information for the trial sponsor
	5c	2	Role of study sponsor and funders, if any, in study design; collection, management, analysis, and interpretation of data; writing of the report; and the decision to submit the report for publication, including whether they will have ultimate authority over any of these activities
	5d	9	Composition, roles, and responsibilities of the coordinating centre, steering committee, endpoint adjudication committee, data management team, and other individuals or groups overseeing the trial, if applicable (see Item 21a for data monitoring committee)

For peer review only

**Introduction**			
Background and rationale	6a	7-8	Description of research question and justification for undertaking the trial, including summary of relevant studies (published and unpublished) examining benefits and harms for each intervention
	6b	n/a	Explanation for choice of comparators
Objectives	7	8	Specific objectives or hypotheses
Trial design	8	9	Description of trial design including type of trial (eg, parallel group, crossover, factorial, single group), allocation ratio, and framework (eg, superiority, equivalence, noninferiority, exploratory)
**Methods: Participants, interventions, and outcomes**			
Study setting	9	10-11	Description of study settings (eg, community clinic, academic hospital) and list of countries where data will be collected. Reference to where list of study sites can be obtained
Eligibility criteria	10	10-11	Inclusion and exclusion criteria for participants. If applicable, eligibility criteria for study centres and individuals who will perform the interventions (eg, surgeons, psychotherapists)
Interventions	11a	n/a	Interventions for each group with sufficient detail to allow replication, including how and when they will be administered
	11b	n/a	Criteria for discontinuing or modifying allocated interventions for a given trial participant (eg, drug dose change in response to harms, participant request, or improving/worsening disease)

For peer review only

	11c	n/a	Strategies to improve adherence to intervention protocols, and any procedures for monitoring adherence (eg, drug tablet return, laboratory tests)
	11d	n/a	Relevant concomitant care and interventions that are permitted or prohibited during the trial
Outcomes	12	9-10	Primary, secondary, and other outcomes, including the specific measurement variable (eg, systolic blood pressure), analysis metric (eg, change from baseline, final value, time to event), method of aggregation (eg, median, proportion), and time point for each outcome. Explanation of the clinical relevance of chosen efficacy and harm outcomes is strongly recommended
Participant timeline	13	10-11	Time schedule of enrolment, interventions (including any run-ins and washouts), assessments, and visits for participants. A schematic diagram is highly recommended (see Figure)
Sample size	14	n/a	Estimated number of participants needed to achieve study objectives and how it was determined, including clinical and statistical assumptions supporting any sample size calculations
Recruitment	15	10-11	Strategies for achieving adequate participant enrolment to reach target sample size
**Methods: Assignment of interventions (for controlled trials)**			
Allocation:			
Sequence generation	16a	n/a	Method of generating the allocation sequence (eg, computer-generated random numbers), and list of any factors for stratification. To reduce predictability of a random sequence, details of any planned restriction (eg, blocking) should be provided in a separate document that is unavailable to those who enrol participants or assign interventions

For peer review only - http://bmjopen.bmj.com/site/about/guidelines.xhtml

Allocation concealment mechanism	16b	n/a	Mechanism of implementing the allocation sequence (eg, central telephone; sequentially numbered, opaque, sealed envelopes), describing any steps to conceal the sequence until interventions are assigned
Implementation	16c	n/a	Who will generate the allocation sequence, who will enrol participants, and who will assign participants to interventions
Blinding (masking)	17a	n/a	Who will be blinded after assignment to interventions (eg, trial participants, care providers, outcome assessors, data analysts), and how
	17b	n/a	If blinded, circumstances under which unblinding is permissible, and procedure for revealing a participant's allocated intervention during the trial
**Methods: Data collection, management, and analysis**			
Data collection methods	18a	10-12	Plans for assessment and collection of outcome, baseline, and other trial data, including any related processes to promote data quality (eg, duplicate measurements, training of assessors) and a description of study instruments (eg, questionnaires, laboratory tests) along with their reliability and validity, if known. Reference to where data collection forms can be found, if not in the protocol
	18b	n/a	Plans to promote participant retention and complete follow-up, including list of any outcome data to be collected for participants who discontinue or deviate from intervention protocols
Data management	19	13	Plans for data entry, coding, security, and storage, including any related processes to promote data quality (eg, double data entry; range checks for data values). Reference to where details of data management procedures can be found, if not in the protocol
Statistical methods	20a	13-14	Statistical methods for analysing primary and secondary outcomes. Reference to where other details of the statistical analysis plan can be found, if not in the protocol

	20b	13	Methods for any additional analyses (eg, subgroup and adjusted analyses)
	20c	n/a	Definition of analysis population relating to protocol non-adherence (eg, as randomised analysis), and any statistical methods to handle missing data (eg, multiple imputation)
**Methods: Monitoring**			
Data monitoring	21a	n/a	Composition of data monitoring committee (DMC); summary of its role and reporting structure; statement of whether it is independent from the sponsor and competing interests; and reference to where further details about its charter can be found, if not in the protocol. Alternatively, an explanation of why a DMC is not needed
	21b	n/a	Description of any interim analyses and stopping guidelines, including who will have access to these interim results and make the final decision to terminate the trial
Harms	22	n/a	Plans for collecting, assessing, reporting, and managing solicited and spontaneously reported adverse events and other unintended effects of trial interventions or trial conduct
Auditing	23	n/a	Frequency and procedures for auditing trial conduct, if any, and whether the process will be independent from investigators and the sponsor
**Ethics and dissemination**			
Research ethics approval	24	14	Plans for seeking research ethics committee/institutional review board (REC/IRB) approval
Protocol amendments	25	n/a	Plans for communicating important protocol modifications (eg, changes to eligibility criteria, outcomes, analyses) to relevant parties (eg, investigators, REC/IRBs, trial participants, trial registries, journals, regulators)
Consent or assent	26a	14	Who will obtain informed consent or assent from potential trial participants or authorised surrogates, and how (see Item 32)

only

For peer review only - http://bmjopen.bmj.com/site/about/guidelines.xhtml

	26b	n/a	Additional consent provisions for collection and use of participant data and biological specimens in ancillary studies, if applicable
Confidentiality	27	14	How personal information about potential and enrolled participants will be collected, shared, and maintained in order to protect confidentiality before, during, and after the trial
Declaration of interests	28	2	Financial and other competing interests for principal investigators for the overall trial and each study site
Access to data	29	16	Statement of who will have access to the final trial dataset, and disclosure of contractual agreements that limit such access for investigators
Ancillary and post-trial care	30	n/a	Provisions, if any, for ancillary and post-trial care, and for compensation to those who suffer harm from trial participation
Dissemination policy	31a	14-15	Plans for investigators and sponsor to communicate trial results to participants, healthcare professionals, the public, and other relevant groups (eg, via publication, reporting in results databases, or other data sharing arrangements), including any publication restrictions
	31b	14-15	Authorship eligibility guidelines and any intended use of professional writers
	31c	14-15	Plans, if any, for granting public access to the full protocol, participant-level dataset, and statistical code
**Appendices**			
Informed consent materials	32	n/a	Model consent form and other related documentation given to participants and authorised surrogates

For peer review only - http://bmjopen.bmj.com/site/about/guidelines.xhtml

Biological specimens	33	n/a	Plans for collection, laboratory evaluation, and storage of biological specimens for genetic or molecular analysis in the current trial and for future use in ancillary studies, if applicable

*It is strongly recommended that this checklist be read in conjunction with the SPIRIT 2013 Explanation & Elaboration for important clarification on the items. Amendments to the protocol should be tracked and dated. The SPIRIT checklist is copyrighted by the SPIRIT Group under the Creative Commons "Attribution-NonCommercial-NoDerivs 3.0 Unported" license.

For peer review only
